# Exploration of the Characteristics of Intestinal Microbiota and Metabolomics in Different Rat Models of Mongolian Medicine

**DOI:** 10.1155/2021/5532069

**Published:** 2021-08-03

**Authors:** Riao Dao, Dongxing Wu, Huan Wang, Habur Jin, Li Li, Xiquan Fu, Chula Sa, Eerdunchaolu

**Affiliations:** ^1^Inner Mongolia University for Nationalities, Mongolian Medical College, Tongliao City, Inner Mongolia Autonomous Region 028000, China; ^2^Department of Psychosomatic Medicine, International Mongolian Medical Hospital of Inner Mongolia Autonomous Region, Hohhot, Inner Mongolia Autonomous Region 010010, China; ^3^Affiliated Hospital of Inner Mongolia University for the Nationalities, Rehabilitation Hospital, Tongliao City, Inner Mongolia Autonomous Region 028000, China; ^4^Second Department of Encephalopathy, Affiliated Hospital of Inner Mongolia University for the Nationalities, Tongliao City, Inner Mongolia Autonomous Region 028000, China

## Abstract

**Background:**

Mongolian medicine is a systematic theoretical system, which is based on the balance among Heyi, Xila, and Badagan. However, the underlying mechanisms remain unclear. This study aimed to explore the characteristics of intestinal microbiota and metabolites in different rat models of Mongolian medicine.

**Methods:**

After establishing rat models of Heyi, Xila, and Badagan, we integrated 16S rRNA gene sequencing and metabolomics.

**Results:**

Heyi, Xila, and Badagan rats had significantly altered intestinal microbial composition compared with rats in the MCK group. They showed 11, 18, and 8 significantly differential bacterial biomarkers and 22, 11, and 15 differential metabolites, respectively. The glucosinolate biosynthesis pathway was enriched only in Heyi rats; the biosynthesis of phenylpropanoids pathway and phenylpropanoid biosynthesis pathway were enriched only in Xila rats; the isoflavonoid biosynthesis pathway, the glycine, serine, and threonine metabolism pathway, and the arginine and proline metabolism pathway were enriched only in Badagan rats.

**Conclusions:**

The intestinal microbiota, metabolites, and metabolic pathways significantly differed among Heyi, Xila, and Badagan rats compared with control group rats.

## 1. Introduction

Traditional Mongolian medicine is an indigenous medicine system widely practiced in China, especially in the Inner Mongolia region [1]. The systematic theoretical system of Mongolian medicine is based on the balance among three roots: Heyi, Xila, and Badagan. Generally, the ratio of three roots in different individuals depends on genetic and environmental factors. An imbalance in these roots results in disease. The Heyi, Xila, and Badagan rat models constructed based on the “Four-Part Medicine Classics” [2] would enhance our understanding of Mongolian medicine. Current research on Mongolian medicine has mainly focused on clinical practice or drugs prescribed in Mongolian medicine [3–5]. However, the underlying mechanisms based on Heyi, Xila, and Badagan are unclear. The digestive system status usually plays an important role in the diagnosis of different diseases in Mongolian medicine, among which the gut environment and intestinal microbiota carry significant importance [6, 7]. Therefore, it is important to understand the underlying pathogenesis of different diseases by comparing the composition of intestinal microbiota in Heyi, Xila, and Badagan rat models.

The intestinal microbiota refers to the various microorganisms present in the gastrointestinal tract, including bacteria, fungi, and viruses [7]. About 3 × 10^6^ genes exist in microbial genome sequences [8]. With the development of next-generation sequencing technology, 16S rRNA sequencing made it possible for us to link intestinal microbiota to various diseases [9]. Dysbiosis is known to cause diseases, such as hypertension [10], inflammatory bowel diseases [11], and type 2 diabetes [12]. To the best of our knowledge, no previous study has linked intestinal microbiota to the different aspects of Heyi, Xila, and Badagan in Mongolian medicine. After oral administration, drugs used in traditional medicine often work by interacting with the intestinal microbiota [9, 13], implying that the intestinal microbiota is important in traditional Chinese medicine and Mongolian medicine. Metabolomics analysis has emerged as an effective tool to study pathogenetic mechanisms [14]. Some recent studies have combined metabolomics analysis with intestinal microbiota analysis [15–17]. A recent study in a twin model demonstrated complex links between host phenotypes and intestinal microbiota based on metabolic profiling [18]. Thus, metabolomics analysis and determination of the composition of intestinal microbiota would help us better understand different diseases in Mongolian medicine.

In the current study, we explored the characteristics of intestinal microbiota and metabolites via an integrated analysis of 16S rRNA sequencing and metabolomics in three Mongolian medicine rat models (Heyi, Xila, and Badagan rat models) to further understand the possible mechanisms underlying diseases related to the three roots in Mongolian medicine. The results would also provide deeper insights into the function of intestinal microbiota.

## 2. Materials and Methods

### 2.1. Rat Model Construction

All rats (10 rats each group) were obtained from Liaoning Changsheng Biotechnology Co. Ltd. (Liaoning, China). The Medical Ethics Committee of the Affiliated Hospital of Inner Mongolia University for The Nationalities approved all experimental procedures (ethic code: NM-LL-2019-12-06-01). There was no intervention put on rats in the control group (MCK). And all rat models were constructed mainly based on the “Four-Part Medicine Classics” [2]. When the rats showed corresponding characteristics of Heyi, Xila, and Badagan described in the “Four-Part Medicine Classics,” the model was considered to be constructed successfully (Supplementary Material 1).

A Heyi rat model was constructed according to the following methods: First, diet intervention, drinking water was replaced by cold black tea (5 g tea + 100 mL distilled water) and the rats were fed buckwheat (8.5 g/day). Second, behavior intervention, the rats were exposed to the continuous cat audio at 70 decibels. Third, Mongolian medicine intervention, the rats were given a dose of 1 mL/100 (g d) Gaburi by gavage. Finally, 0.1 mL tail vein bloodletting was performed on the rats at 5 pm every two days. It took 31 days to construct the Heyi rat model.

A Xila rat model was constructed according to the following methods: First, diet intervention, the rats were given 1 mL liqueur by gavage once every other day, were given 0.7 g/kg fruit oil at 6 am every day, and were fed yellow rice (15 g/day). Second, behavior intervention, the rats were under the environment of 29 ± 2°C. Third, Mongolian medicine intervention, the rats were given 0.7 g/kg pepper by gavage daily at 12 noon. It took 21 days to construct the Xila rat model.

A Badagan rat model was constructed according to the following methods: First, diet intervention, the rats were fed lard and wheat flour (mixed in a ratio of 1 : 4). Second, behavior intervention, the rats were under the environment of 60 ± 5% humidity. Third, Mongolian medicine intervention, the rats were given 4 mL dandelion (200% decoction) by gavage. It took 49 days to construct the Badagan rat model.

### 2.2. Fecal Sample Collection and DNA Extraction

Fecal samples were collected in a sterile conical tube and stored at −80°C. According to the manufacturer's instruction, the DNA was extracted using an E.Z.N.A. feces DNA kit (Omega Bio-Tek, Norcross, GA, USA). The DNA quality was determined by 1% agarose gel electrophoresis, and the DNA concentration was determined using a NanoDrop 2000 spectrophotometer (Thermo Fisher Scientific, USA).

### 2.3. 16S rRNA Microbial Community Analysis

The primer 341F (5′-CCTAYGGGRBGCASCAG-3′) 806R (5′-GGACTACNNGGGTATCTAAT-3′) was used to amplify the bacterial 16S rRNA gene V3-V4 region, which was performed on the Illumina HiSeq sequencing platform (Illumina, USA). As for the paired-end sequences obtained, the primer adapter sequences were removed, and various samples were distinguished based on the barcode tag sequences. And the valid data of the samples were obtained after the quality control filtering. FLASH software (version 1.2.11) [19] was used to splice the paired-end sequences, and Trimmomatic software (version 0.33) [20] was used to filter the spliced sequences. UCHIME software (version 8.1) [21] was used to remove the chimera sequences in order to obtain valid data for further analysis. Based on 97% similarity, all sequences were clustered in operational taxonomic units (OTU) using USEARCH software (version 10.0) [22], which was filtered with 0.005% of all sequences as a threshold. In order to determine the classification, RDP Classifier software (version 2.2) (http://rdp.cme.msu.edu/classifier/classifier.jsp) [23] was used to compare the representative sequence of each OTU with the Silva database (https://www.arb-silva.de/) [24]. The alpha diversity of microbiota was calculated using mothur software (version 1.30) [25], including Chao1, ACE, Shannon index, and Simpson index. The *β* diversity was estimated according to the Bray Curtis distance algorithm and then was visualized using nonmetric multidimensional scaling (NMDS). The differential biomarkers between different groups were found according to the linear discriminant analysis effect size (LEfSe) [26] based on relative abundance. The KEGG function of microbiota was predicted using Phylogenetic Investigation of Communities by Reconstruction of Unobserved States (PICRUSt) software (version 1.1.4) [27].

### 2.4. Serum Sample Preparation for Metabolome

Blood samples were collected from the abdominal aorta, and the serum was separated and stored at −80°C. Then, 200 *μ*L of the serum was taken and 3 times volume of precooled acetonitrile solution was added. The sample was vortexed and mixed, and then it was placed in a refrigerator at −20°C for 30 min. Subsequently, the sample was centrifuged (14000 g, 4°C, 15 min) and the supernatant was transferred in a new centrifuge tube for concentration and drain. The 1 : 1 (v/v) mixture of mobile phase A (ammonium acetate) and mobile phase B (acetonitrile) was used to redissolve the sample; after the high-speed centrifugation, the sample was used for HPLC-MS analysis. The target compounds were separated on an Accucore Hilic (100 × 2.1 mm, 2.6 *μ*m) liquid chromatography column, using Vanquish (Thermo Fisher Scientific) ultra-performance liquid chromatography. And the liquid chromatography consisted of 10 mM ammonium acetate as phase A and acetonitrile/10 mM ammonium acetate as phase B (9 : 1). Gradient elution was used: 0∼1 min, 100% A; 1∼9 min, 0%∼100% B; 9∼12 min, 100% B; and 12.1∼15 min, 100% A. The flow rate of mobile phase was 0.35 mL/min; the column temperature was 35°C; the sample tray temperature was 4°C; and the injection volume was 2 *μ*L.

### 2.5. Metabolite Analysis

The serum metabolites were analyzed using Thermo Scientific's Q Exactive mass spectrometer. The positive and negative ions were scanned once each. The positive ion scan was performed firstly, after which, the negative ion scan was performed. The full scan range was 70–1050 m/z. In the full scan, the precursor ions with TOP10 ion intensity were selected for secondary MS identification. The precursor ion was fragmented according to the HCD method, which was used for secondary mass spectrometry sequence determination and then generated the mass spectrometry detection original file. Subsequently, the raw data were transformed in mzML format using ProteoWizard software, and XCMS was used to perform retention time correction, peak identification, peak extraction, peak integration, peak alignment, etc. Then, identification of metabolites was based on the Compound Discover V3.0 (CD) and mzCloud database. SIMCA-P software was used for orthogonal partial least square discriminate analysis (OPLS-DA). In order to select different variables as potential markers, VIP-plot (VIP >1) was obtained from OPLS analysis. The differential metabolites with VIP >1 and *P* value < 0.05 were screened.

The OPLS-DA model was validated using 7-fold cross-validation. Then, *R*^2^*Y* (model explainability of the categorical variable *Y*) and *Q*^2^ (predictability of model) were used to determine the validity of the model. Finally, the permutation test was performed to further test the validity of the model, which was done via randomly changing the arrangement of categorical variable *Y* (*n* = 200 times) and obtaining random *Q*^2^ values.

### 2.6. Statistical Analysis

The Wilcoxon rank-sum test (R software v3.6.2) was used to compare the *α* diversity (ACE index, Chao1 index, Shannon index, and Simpson index) and microbiota between various groups, and *P* < 0.05 was considered as the significance threshold. Analysis of similarities (ANOSIM) was used to analyze the differences between and within groups. The Kruskal–Wallis sum-rank test was used to determine the alterations in abundance between different groups in LEfSe analysis, and |LDA score| > 3 and *P* < 0.05 were taken as the difference screening thresholds.

## 3. Results

### 3.1. Changes in Intestinal Microbiota Diversity among Different Mongolian Medicine Rat Models

Based on the results of 16S rRNA sequence analysis, the changes of intestinal microbiota of three different Mongolian medicine rat models and control group rats were investigated. The results were clustered in operational taxonomic units (OTU) based on over 97% similarity. The rarefaction curves, based on the number of sample reads and OTUs, tended to be flat (Figure S1), indicating that the amount of sequencing data was sufficient to reflect the species diversity in all samples. The ACE, Chao1, Shannon, and Simpson indexes were used to evaluate microbial alpha diversity.

**Figure 1:**
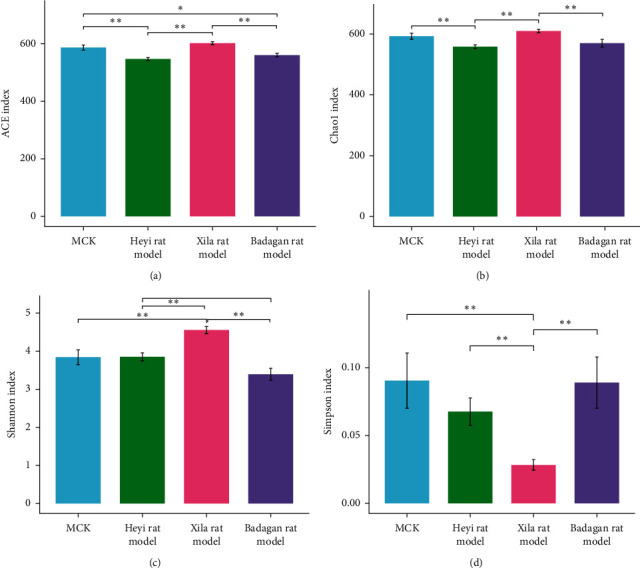
The alpha diversity analysis of intestinal microbiota community. (a) ACE index. (b) Chao1 index. (c) Shannon index. (d) Simpson index. *X* axis: different groups; *Y* axis: the corresponding diversity index. The Wilcoxon test was used to determine the significance of differences between any two groups. Statistical significance: ^*∗*^*P* < 0.05, ^*∗∗*^*P* < 0.01, ^*∗∗∗*^*P* < 0.001, and ^*∗∗∗∗*^*P* < 0.0001.

Compared with the MCK group, the ACE and Chao1 indexes of intestinal microbiota in the Heyi rat model significantly decreased (*P* value < 0.05), but the Shannon and Simpson indexes did not significantly differ. Compared with the MCK group, the ACE and Chao1 indexes of intestinal microbiota in the Xila rat model did not significantly differ, but the Shannon index increased significantly and the Simpson index decreased significantly. Compared with the MCK group, the ACE index of intestinal microbiota in the Badagan rat model significantly decreased, but the Chao1, Shannon, and Simpson indexes did not significantly differ (Figures 1(a)–1(d)). The results showed that compared with the MCK group, the richness of intestinal microbiota in Heyi rats and Badagan rats was decreased, but the diversity did not significantly differ; however, the richness of intestinal microbiota in Xila rats did not significantly differ, but the diversity increased.

Compared with Heyi and Badagan rat models, the ACE, Chao1, and Shannon indexes of intestinal microbiota in the Xila rat model significantly increased, but the Simpson index decreased significantly. Compared with the Heyi rat model, the Shannon index of intestinal microbiota in the Badagan rat model decreased significantly, but the ACE, Chao1, and Simpson indexes did not significantly differ (Figures 1(a)–1(d)). The results above indicated that the richness and diversity of intestinal microbiota in Xila rats both increased significantly compared with Heyi and Badagan rats. Moreover, compared with Heyi rats, the richness of intestinal microbiota in Badagan rats did not significantly differ, but the diversity decreased significantly.

**Figure 2:**
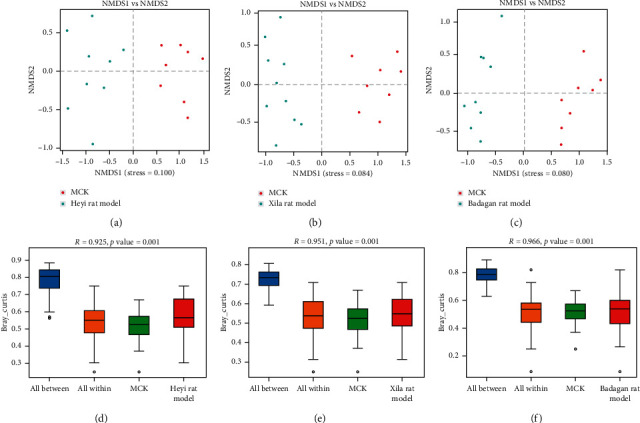
The beta diversity analysis of intestinal microbiota community. (a) The NMDS of the Heyi rat model and MCK group. The dots: samples; colors: different groups. The closer the dots, the more similar the species compositions. (b) The NMDS of the Xila rat model and MCK group. (c) The NMDS of the Badagan rat model and MCK group. (d) ANOSIM analysis of the Heyi rat model and MCK group. *Y* axis: beta distance. All between: beta distance of samples in all groups; all within: beta distance of samples within the group. *P* value < 0.05 was considered as significant difference. *R* > 0 indicated that the difference between groups was greater than the difference within the groups; *R* < 0 indicated that the difference within the group is greater than the difference between the groups; the greater the |R| value, the greater the relative difference. (e) ANOSIM analysis of the Xila rat model and MCK group. (f) ANOSIM analysis of the Badagan rat model and MCK group.

According to the results of *β* diversity analysis, significant differences were noted between rats in the Heyi and MCK groups (Figures 2(a) and 2(d); *R* = 0.925, *P* = 0.001), the Xila and MCK groups (Figures 2(b) and 2(e); *R* = 0.951, *P*= 0.001), and the Badagan and MCK groups (Figures 2(c) and 2(f); *R* = 0.966, *P* = 0.001). These results showed that the *β* diversity of intestinal microbiota of all three models was significantly different from that of the MCK group. Collectively, the intestinal microbial structure of Heyi, Xila, and Badagan rats significantly differed from that of the control group rats.

### 3.2. Alterations of the Intestinal Microbiota Composition in Different Mongolian Medicine Rat Models

**Figure 3:**
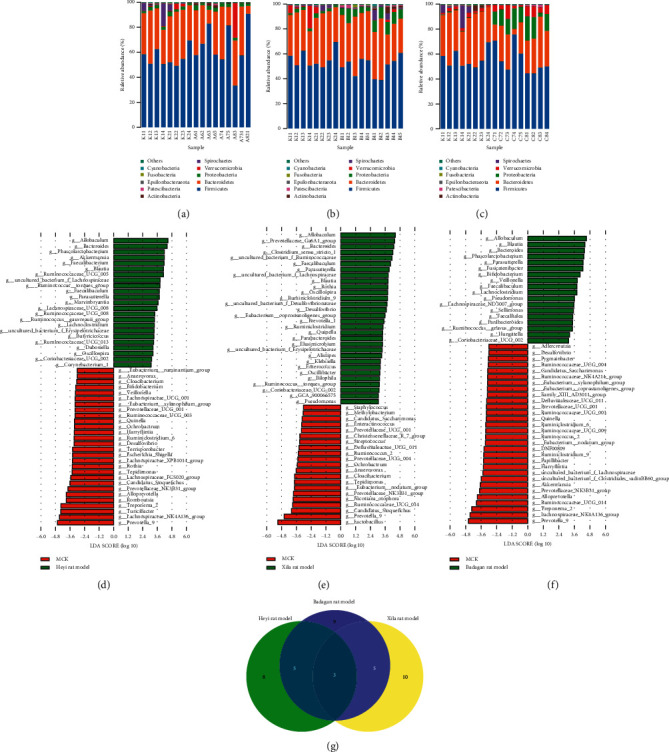
Alterations of intestinal microbiota composition. (a) Compared with the MCK group, the abundance changes of microbiota from the phylum level in the Heyi rat model. (b) Compared with the MCK group, the abundance changes of microbiota from the phylum level in the Xila rat model. (c) Compared with the MCK group, the abundance changes of microbiota from the phylum level in the Badagan rat model. (d) The significantly differential biomarkers between the Heyi rat model and MCK group based on LEfSe analysis. (e) The significantly differential biomarkers between the Xila rat model and MCK group based on LEfSe analysis. (f) The significantly differential biomarkers between the Badagan rat model and MCK group based on LEfSe analysis. (g) Venn diagram of significantly differential biomarkers.

In all rats from three models, Firmicutes and Bacteroidetes accounted for 80% of the top 10 bacteria making up the intestinal microbiota at the phylum level (Figures 3(a)–3(c)). Compared with the MCK group, the abundance of Verrucomicrobia in the Heyi rat model significantly increased (*P* = 0.011), whereas the abundance of Spirochaetes (*P* value = 8.2*e* − 5), Patescibacteria (*P* value = 0.046), and Cyanobacteria (*P* value = 8.2*e* − 5) decreased significantly in the Heyi rat model (Table S1). Compared with the MCK group, the abundance of Proteobacteria (*P* value = 9.1*e* − 5) and Elusimicrobia (*P* value = 4*e* − 5) increased significantly in the Xila rat model (Table S2). Compared with the MCK group, the abundance of Actinobacteria (*P* value = 8.2*e* − 5) and Proteobacteria (*P* value = 0.027) increased significantly in the Badagan rat model, whereas the abundance of Bacteroidetes (*P* value = 0.027), Spirochaetes (*P* value = 8.2*e* − 5), Verrucomicrobia (*P* value = 0.0055), and Patescibacteria (*P* value = 8.2*e* − 5) significantly decreased (Table S3).

To investigate the specific intestinal bacterial biomarkers at the genus level, line discriminant analysis (LDA) effect size (LEfSe) analysis was performed on all three different Mongolian medicine rat models. The bacteria abundance of 23 genera in the Heyi rat model was significantly higher than that in MCK rats (LDA >3, *P* value < 0.05) (Figure 3(d)). The bacteria abundance of 30 genera in the Xila rat model was significantly higher than that in MCK rats (LDA >3, *P* value < 0.05) (Figure 3(e)). The bacteria abundance of 18 genera in the Badagan rat model was significantly higher than that in MCK rats (LDA >3, *P* value < 0.05) (Figure 3(f)). The abundance of *Allobaculum*, *Parasutterella*, *Coriobacteriaceae_UCG_002*, *Faecalibaculum*, *Bacteroides*, and *Blautia* was high in three models. The abundance of 11 bacteria was only higher in the Heyi rat model, including *Faecalibacterium*, Ruminococcus__gauvreauii_group, *Ruminococcaceae_UCG_008*, *Marvinbryantia*, *Akkermansia*, Ruminococcaceae_UCG_005, Lachnospiraceae_UCG_008, Ruminococcaceae_UCG_013, *Dubosiella*, *Butyricicoccus*, and Corynebacterium_1. The abundance of 18 bacteria was higher only in the Xila rat model, including *Alistipes*, uncultured_bacterium_f_Desulfovibrionaceae, *Elusimicrobium*, *Bilophila*, Prevotella_1, GCA_900066575, Clostridium_sensu_stricto_1, Prevotellaceae_Ga6A1_group, *Rothia*, *Quinella*, *Desulfovibrio*, Ruminiclostridium_9, Eubacterium__coprostanoligenes_group, *Klebsiella*, *Oscillibacter*, uncultured_bacterium_f_Ruminococcaceae, *Enterococcus*, and *Ruminiclostridium*. The abundance of 8 bacteria was higher only in the Badagan rat model, including *Hungatella*, *Sellimonas*, *Faecalitalea*, *Fusicatenibacter*, Ruminococcus__gnavus_group, *Bifidobacterium*, *Veillonella*, and Lachnospiraceae_ND3007_group (Figure 3(g)).

### 3.3. Prediction of the Function of Intestinal Microbiota in Different Mongolian Medicine Rat Models

**Figure 4:**
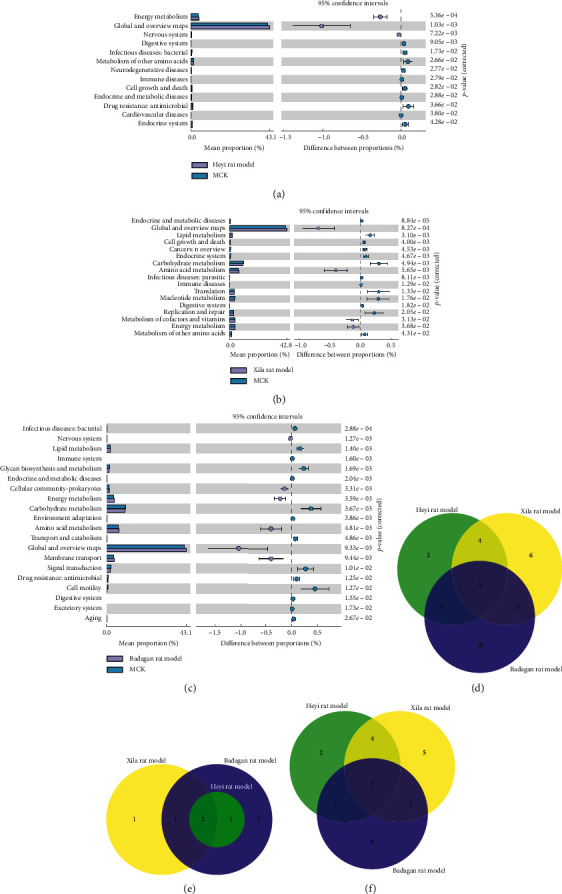
KEGG functional prediction of intestinal microbiota. (a) KEGG functional prediction of the Heyi rat model and MCK group. The left side shows the abundance ratio of different pathways in two sets of samples; the right side shows the *P* value. (b) KEGG functional prediction of the Xila rat model and MCK group. (c) KEGG functional prediction of the Badagan rat model and MCK group. (d) Venn diagram of significantly different KEGG pathways between the experimental groups and control group. (e) Venn diagram of significantly increased KEGG pathways in the experimental groups. (f) Venn diagram of significantly decreased KEGG pathways in the experimental groups.

To predict the function of intestinal microbiota in different models, PICRUSt was used. The results of KEGG function analysis of three models are displayed in Figures 4(a)–4(c). Compared with the MCK group, the abundance of 3 KEGG pathways significantly increased and that of 10 pathways significantly decreased in the Heyi rat model, total 13 KEGG pathways were significantly different between Heyi and MCK groups (Figure 4(a)). Compared with the MCK group, the abundance of 4 KEGG pathways significantly increased and that of 13 pathways significantly decreased in the Xila rat model, and 17 KEGG pathways were significantly different between Xila and MCK groups (Figure 4(b)). Compared with the MCK group, the abundance of 6 KEGG pathways significantly increased and that of 14 pathways decreased significantly in the Badagan rat model, and 20 KEGG pathways were significantly different between the Badagan and MCK groups (Figure 4(c)).

Moreover, the abundance of 4 KEGG pathways was significantly different between all three experimental groups (Heyi, Xila, and Badagan rat models) and the control group (Figure 4(d)), among which the abundance of “global and overview maps” pathway and “energy metabolism” pathway was significantly increased in the experimental groups (Figure 4(e)), but that of “digestive system” pathway and “endocrine and metabolic diseases” pathway was significantly reduced (Figure 4(f)).

Furthermore, the abundance of some pathways was only changed in one certain model. The abundance of “cardiovascular diseases” pathway and “neurodegenerative diseases” pathway was significantly decreased only in the Heyi rat model. The abundance of “metabolism of cofactors and vitamins” pathway was significantly increased and the abundance of “nucleotide metabolism” pathway, “replication and repair” pathway, “infectious diseases: parasitic” pathway, and “cancers: overview translation” pathway was decreased only in the Xila rat model. The abundance of “cellular community-prokaryotes” pathway was significantly increased and the abundance of “transport and catabolism” pathway, “glycan biosynthesis and metabolism” pathway, “cell motility” pathway, “environmental adaptation” pathway, “excretory system” pathway, “aging” pathway, “immune system” pathway, “signal transduction” pathway, and “membrane transport” pathway was decreased only in the Badagan rat model. Our data implied that the abundance of various pathways has been altered in different models compared with the MCK group.

### 3.4. Identification of Serum Metabolic Profile and Metabolic Markers in Different Mongolian Medicine Rat Models

**Figure 5:**
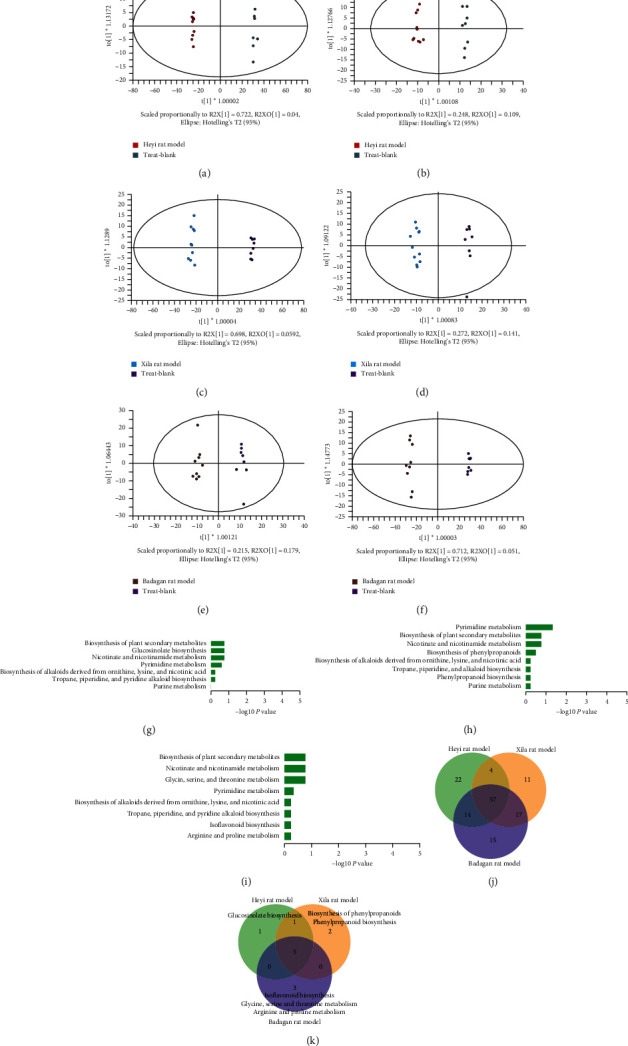
Serum metabolic profile of all rats. (a, b) The OPLS-DA score plot of the Heyi rat model and MCK group on positive ion and negative ion modes. (c, d) The OPLS-DA score plot of the Xila rat model and MCK group on positive ion and negative ion modes. (e, f) The OPLS-DA score plot of the Badagan rat model and MCK group on positive ion and negative ion modes. (g) Differential metabolites between the Heyi rat model and MCK group were enriched in 7 KEGG pathways. (h) Differential metabolites between the Xila rat model and MCK group were enriched in 8 KEGG pathways. (i) Differential metabolites between the Badagan rat model and MCK group were enriched in 8 KEGG pathways. (j) Venn diagram of the differential metabolites between Heyi rat model vs. MCK, Xila rat model vs. MCK, and Badagan rat model vs. MCK. (k) Venn diagram of enriched KEGG pathways of differential metabolites between Heyi rat model vs. MCK, Xila rat model vs. MCK, and Badagan rat model vs. MCK.

According to the results of metabolite profile analysis, Heyi, Xila, and Badagan rat models presented significant differences compared with the MCK group (Figures 5(a)–5(f)).

In the OPLS-DA multivariate model, metabolites with VIP scores >1 and *P* value < 0.05 were considered as differential metabolites. Compared with the MCK group, 30 differential metabolites were detected in the Heyi rat model after positive ion scan and 64 differential metabolites were noted after negative ion scan (Table S4), resulting in 94 differential metabolites involved in 7 metabolic pathways (Figure 5(g)). Compared with the MCK group, 35 differential metabolites were detected in the Xila rat model after positive ion scan and 51 differential metabolites were noted after negative ion scan (Table S5), resulting in 86 differential metabolites involved in 8 metabolic pathways (Figure 5(h)). Compared with the MCK group, 37 differential metabolites were detected in the Badagan rat model after positive ion scan and 63 differential metabolites were noted after negative ion scan (Table S6), resulting in 100 differential metabolites involved in 8 metabolic pathways (Figure 5(i)).

In addition, 22, 11, and 15 differential metabolites were detected only in the Heyi, Xila, and Badagan rat models, respectively (Figure 5(j), Table S7). Moreover, the “glucosinolate biosynthesis” pathway was enriched only in the Heyi rat model; the “biosynthesis of phenylpropanoids” pathway and “phenylpropanoid biosynthesis” pathway were enriched only in the Xila rat model; the “isoflavonoid biosynthesis” pathway, “glycine, serine, and threonine metabolism” pathway, and “arginine and proline metabolism” pathway were enriched only in the Badagan rat model.

## 4. Discussion

We explored the characteristics of intestinal microbiota and metabolomics in different Mongolian medicine rat models. In this study, a joint analysis of 16S rRNA gene sequencing and metabolomics was conducted to investigate the potential mechanisms underlying diseases related to Heyi, Xila, and Badagan. Our results indicated that the intestinal microbiota of Heyi, Xila, and Badagan rat models significantly differed from that of control rats. Metabolites and metabolic pathways in Heyi, Xila, and Badagan rats were also significantly different from those in control rats.

The alpha and beta diversity and intestinal microbiota composition were investigated, as the link between intestinal microbiota changes and many metabolic diseases have been reported [28, 29]. Our results showed that compared with the MCK group, the alpha diversity of intestinal microbiota in Xila rats was increased, but that in Heyi and Badagan rats showed no significant difference. The alpha diversity of intestinal microbiota in Xila rats was also higher than that in Heyi and Badagan rats. The results of beta diversity indicated that there was a significant dissimilarity between the control group and Mongolian medicine rat models. The intestinal microbiota of Heyi, Xila, and Badagan rats was significantly altered compared with that of control group rats, which was probably an important contributor to different diseases in Mongolian medicine. Furthermore, intestinal microbiota composition was also investigated. Firmicutes and Bacteroidetes were found to be the dominant strains in all rats, consistent with former studies reporting that Firmicutes and Bacteroidetes are two main phyla comprising gut microbiota in healthy humans [30]. Moreover, the abundance of some phyla in our disease models was significantly different from that in control group rats. Verrucomicrobia, usually colonized in the mucosal layer, is considered a promising probiotics [31, 32]. The abundance of Verrucomicrobia was increased in Heyi rats but decreased in Badagan rats compared with control group rats, which might be responsible for the contrary manifestations of Heyi disease and Badagan disease. The abundance of Proteobacteria was increased both in Xila and Badagan rats, and it is reported that the increase in abundance of Proteobacteria resulted in an imbalanced gut microbiota composition and consequently metabolic disorders [33]. Furthermore, we found that bacteria biomarkers of some genera existed specifically in certain disease in Mongolian medicine. There were 11, 18, and 8 bacterial biomarkers that were increased only in Heyi, Xila, and Badagan rats, respectively. Some of these bacterial genera have been associated with diseases, such as *Faecalibacterium* [34], Prevotella_1 [35], Prevotellaceae [36], *Alistipes* [37], *Bilophila* [38], *Hungatella* [39], and *Sellimonas* [40], which might contribute to diseases in Mongolian medicine. In addition, the differential KEGG pathways were found between Mongolian medicine rat models and control group rats. The abundance of “global and overview maps” pathway and “energy metabolism” pathway was significantly increased in the disease models, but the abundance of “digestive system” pathway and “endocrine and metabolic diseases” pathway was significantly decreased. We suspected that these pathways might play an essential role in three Mongolian medicine rat models. However, further studies should be conducted to further understand the intestinal microbiota in different diseases in Mongolian medicine.

Furthermore, investigations of metabolites and metabolic pathways in Heyi, Xila, and Badagan rats revealed 22, 11, and 15 differential metabolites specific to Heyi, Xila, and Badagan rats, respectively. The “glucosinolate biosynthesis” pathway was enriched only in Heyi rats, whereas the “biosynthesis of phenylpropanoids” pathway and “phenylpropanoid biosynthesis” pathway were enriched only in Xila rats. The “isoflavonoid biosynthesis” pathway, “glycine, serine, and threonine metabolism” pathway, and “arginine and proline metabolism” pathway were enriched only in Badagan rats. These specific metabolic pathways probably play an important role in the characteristics of the various Mongolian medicine rat models. Moreover, we noticed that most of these pathways were related to the metabolism of certain amino acids, which might be correlated with the diseases in Mongolian medicine. For example, it has been reported that glycine-conjugated metabolites were involved in chronic kidney disease and hypertension in rats [41]. Gut-derived D-serine has renoprotective effects on the kidney in acute kidney injury [42]. L-arginine protects the intestinal barrier by promoting expression of tight junction proteins in rats [43]. Therefore, the kidney and gut are probably more important for the manifestations of diseases in Mongolian medicine. Collectively, although metabolomics analysis helps us better understand the three disease aspects in Mongolian medicine, the specific role of each pathway in various diseases in Mongolian medicine remains to be further studied.

In spite of this, there are still several limitations in our present study. First, only 16S rRNA sequencing and metabolomics were included in our research, which might lead to inevitable deviation in the results. Multiple omics data like methylation data could be further studied in the future. Moreover, the detailed underlying reasons for the pathway changes should be further explored.

## 5. Conclusions

In conclusion, we have firstly explored the characteristics of intestinal microbiota and metabolomics in different Mongolian medicine rat models by integrating 16S rRNA sequencing and metabolomics approaches. Our data showed that the intestinal microbiota, metabolites, and metabolic pathways in Heyi, Xila, and Badagan rats were significantly different from those in control group rats. Despite the detailed mechanisms remain to be clarified, our research has provided more reference information for diseases in Mongolian medicine.

## Data Availability

The data sets of this study are available on request to the corresponding author.

## Additional Points

*Highlights*. (1) Compared with the MCK group, the intestinal microbial structure of Heyi rats, Xila rats, and Badagan rats was significantly altered. (2) Metabolites and metabolic pathways in Heyi, Xila, and Badagan rats were also significantly different from those in control group rats.

## Supplementary Materials

Supplementary Materials Figure S1: the rarefaction curves of all samples. Table S1: relative abundance of microbial phylum (percentage) in the Heyi rats and control rats. Table S2: relative abundance of microbial phylum (percentage) in the Xila rats and control rats. Table S3: relative abundance of microbial phylum (percentage) in the Badagan rats and control rats. Table S4: differential metabolites of Heyi rat samples compared with control group. Table S5: differential metabolites of Xila rat samples compared with control group. Table S6: differential metabolites of Badagan rat samples compared with control group. Table S7: differential metabolites only present in a group of rats.
