# Supplementary material for: Exploration of the Characteristics of Intestinal Microbiota and Metabolomics in Different Rat Models of Mongolian Medicine
Source: Evid Based Complement Alternat Med. 2021 Aug 3;2021:5532069. doi: 10.1155/2021/5532069 (PMC8356010; doi:10.1155/2021/5532069)
Supplement: Supplementary Materials — Figure S1: the rarefaction curves of all samples. Table S1: relative abundance of microbial phylum (percentage) in the Heyi rats and control rats. Table S2: relative abundance of microbial phylum (percentage) in the Xila rats and control rats. Table S3: relative abundance of microbial phylum (percentage) in the Badagan rats and control rats. Table S4: differential metabolites of Heyi rat samples compared with control group. Table S5: differential metabolites of Xila rat samples compared with control group. Table S6: differential metabolites of Badagan rat samples compared with control group. Table S7: differential metabolites only present in a group of rats. [file 5532069.f1.zip › 5532069.f1/Supplementary material 1.docx]

**Supplementary material 1**

All rat models were constructed mainly based on the “Four-Part Medicine Classics” (https://wiki.cnki.com.cn/HotWord/2150531.htm). When the rats showed corresponding characteristics of Heyi, Xila and Badagan described in “Four-Part Medicine Classics”, the model was considered to be constructed successfully. And the detailed basis of different rat models mainly referred to the following information.

**1. Heyi rat model:**

It results from multiple external causes, including abnormal climate change, lack of nutrition for a long time, eating more bitter, astringent, or rough foods (such as buckwheat, gaburi), physical consuming in a hungry state, mental stimulation (like insomnia, panic or sorrow), and hemorrhage.

**The model is considered to be constructed successfully when the following symptoms appear:**

Hyperactivity, insomnia (less sleep time), rough skin and tongue coating, chills and trembling, liking warmth, clear and foamy urine.

**2. Xila rat model**:

It results from multiple external causes. In terms of climate, the weather is overheated or not cold. In terms of diet, the patients may usually have salty and pungent foods, such as wine, chili, sauce, vinegar, long-lived meat products, pepper and so on. In terms of daily life, they are often surrounded in hot and dry environment.

**The model is considered to be constructed successfully when the following symptoms appear:**

Acute onset, rapid disease course, dry mouth, red eyes, easy to diarrhea, red-yellow and strong smell urine.

**3. Badagan rat model**:

It results from multiple external causes, including laziness, abnormal climate change such as rainy and cool summer, excessive cold in winter, eating more undigestible foods (like fatty meat, oil) and bitter, sweet, astringent, greasy, cool, heavy diets (such as dandelion).

**The model is considered to be constructed successfully when the following symptoms appear:**

Slow onset, long disease course, moist and soft tongue, fat, slowly respond, sleepy, cool body, light color and less smell urine.
