# Supplementary material for: Exploration of the Characteristics of Intestinal Microbiota and Metabolomics in Different Rat Models of Mongolian Medicine
Source: Evid Based Complement Alternat Med. 2021 Aug 3;2021:5532069. doi: 10.1155/2021/5532069 (PMC8356010; doi:10.1155/2021/5532069)
Supplement: Supplementary Materials — Figure S1: the rarefaction curves of all samples. Table S1: relative abundance of microbial phylum (percentage) in the Heyi rats and control rats. Table S2: relative abundance of microbial phylum (percentage) in the Xila rats and control rats. Table S3: relative abundance of microbial phylum (percentage) in the Badagan rats and control rats. Table S4: differential metabolites of Heyi rat samples compared with control group. Table S5: differential metabolites of Xila rat samples compared with control group. Table S6: differential metabolites of Badagan rat samples compared with control group. Table S7: differential metabolites only present in a group of rats. [file 5532069.f1.zip › 5532069.f1/Table S1-V2.docx]

Table S1 Relative abundance of microbial phylum (percentage) in the Heyi rat model and control rats

| **Items** | **MCK** | **Heyi rat model** | **Pvalue** |
| --- | --- | --- | --- |
| Firmicutes | 0.5588±0.0706 | 0.6495±0.1776 | 0.2 |
| Bacteroidetes | 0.3532±0.0608 | 0.2751±0.1335 | 0.32 |
| Proteobacteria | 0.0233±0.0079 | 0.02628±0.0082 | 0.37 |
| Verrucomicrobia | 0.0112±0.0149 | 0.0345±0.0925 | 0.011 |
| Spirochaetes | 0.0421±0.0617 | 0.0002±0.0001 | 8.20E-05 |
| Actinobacteria | 0.0038±0.0022 | 0.0087±0.0076 | 0.24 |
| Patescibacteria | 0.0021±0.0013 | 0.0017±0.0039 | 0.046 |
| Epsilonbacteraeota | 0.0011±0.0007 | 0.0009±0.0010 | 0.14 |
| Fusobacteria | 0.001±0.0007 | 0.0009±0.0003 | 0.67 |
| Cyanobacteria | 0.0014±0.001 | 0.0003±0.0000 | 8.20E-05 |
