# Supplementary material for: Exploration of the Characteristics of Intestinal Microbiota and Metabolomics in Different Rat Models of Mongolian Medicine
Source: Evid Based Complement Alternat Med. 2021 Aug 3;2021:5532069. doi: 10.1155/2021/5532069 (PMC8356010; doi:10.1155/2021/5532069)
Supplement: Supplementary Materials — Figure S1: the rarefaction curves of all samples. Table S1: relative abundance of microbial phylum (percentage) in the Heyi rats and control rats. Table S2: relative abundance of microbial phylum (percentage) in the Xila rats and control rats. Table S3: relative abundance of microbial phylum (percentage) in the Badagan rats and control rats. Table S4: differential metabolites of Heyi rat samples compared with control group. Table S5: differential metabolites of Xila rat samples compared with control group. Table S6: differential metabolites of Badagan rat samples compared with control group. Table S7: differential metabolites only present in a group of rats. [file 5532069.f1.zip › 5532069.f1/Table S2-v2.docx]

Table S2 Relative abundance of microbial phylum (percentage) in the Xila rat model and control rats

| **Items** | **MCK** | **Xila rat model** | **Pvalue** |
| --- | --- | --- | --- |
| Firmicutes | 0.5588±0.0706 | 0.5036±0.0747 | 0.32 |
| Bacteroidetes | 0.3532±0.0608 | 0.3634±0.0849 | 0.83 |
| Proteobacteria | 0.0233±0.0079 | 0.0771±0.0268 | 9.10E-05 |
| Spirochaetes | 0.0421±0.0617 | 0.0046±0.0083 | 0.055 |
| Verrucomicrobia | 0.0112±0.0149 | 0.0185±0.0266 | 0.97 |
| Epsilonbacteraeota | 0.0011±0.0007 | 0.0117±0.0163 | 0.055 |
| Actinobacteria | 0.0037±0.0022 | 0.0087±0.0072 | 0.083 |
| Elusimicrobia | 0.0005±0.0004 | 0.0046±0.0057 | 4.00E-05 |
| Tenericutes | 0.0009±0.0008 | 0.0029±0.0064 | 0.97 |
| Cyanobacteria | 0.0015±0.0010 | 0.0022±0.003 | 0.83 |
