# Supplementary material for: Exploration of the Characteristics of Intestinal Microbiota and Metabolomics in Different Rat Models of Mongolian Medicine
Source: Evid Based Complement Alternat Med. 2021 Aug 3;2021:5532069. doi: 10.1155/2021/5532069 (PMC8356010; doi:10.1155/2021/5532069)
Supplement: Supplementary Materials — Figure S1: the rarefaction curves of all samples. Table S1: relative abundance of microbial phylum (percentage) in the Heyi rats and control rats. Table S2: relative abundance of microbial phylum (percentage) in the Xila rats and control rats. Table S3: relative abundance of microbial phylum (percentage) in the Badagan rats and control rats. Table S4: differential metabolites of Heyi rat samples compared with control group. Table S5: differential metabolites of Xila rat samples compared with control group. Table S6: differential metabolites of Badagan rat samples compared with control group. Table S7: differential metabolites only present in a group of rats. [file 5532069.f1.zip › 5532069.f1/Table S4-v2.docx]

Table S4 Differential metabolites of Heyi rat model samples compared with control group

| **NO** | **VIP** | **Name** | **Formula** | **Model** | **RT [min]** | **P** |
| --- | --- | --- | --- | --- | --- | --- |
| 1 | 1.14367 | PEG n8 | C16 H34 O9 | Positiveion | 4.546 | 2.14E-11 |
| 2 | 1.13681 | PEG n7 | C14 H30 O8 | Positiveion | 4.42 | 4.99E-10 |
| 3 | 1.11381 | PEG n6 | C12 H26 O7 | Positiveion | 4.28 | 3.49E-08 |
| 4 | 1.08844 | PEG n5 | C10 H22 O6 | Positiveion | 4.118 | 2.07E-07 |
| 5 | 1.15796 | PEG n16 | C32 H66 O17 | Positiveion | 5.233 | 1.00E-17 |
| 6 | 1.1559 | PEG n15 | C30 H62 O16 | Positiveion | 5.17 | 9.95E-15 |
| 7 | 1.15622 | PEG n14 | C28 H58 O15 | Positiveion | 5.102 | 8.28E-16 |
| 8 | 1.15856 | PEG n13 | C26 H54 O14 | Positiveion | 5.027 | 5.65E-19 |
| 9 | 1.15804 | PEG n12 | C24 H50 O13 | Positiveion | 4.776 | 3.36E-18 |
| 10 | 1.15742 | PEG n11 | C22 H46 O12 | Positiveion | 4.871 | 8.54E-16 |
| 11 | 1.15629 | PEG n10 | C20 H42 O11 | Positiveion | 4.558 | 2.78E-16 |
| 12 | 1.14691 | Nootkatone | C15 H22 O | Positiveion | 6.604 | 1.55E-14 |
| 13 | 1.15783 | N,N-Diethylthiourea | C5 H12 N2 S | Positiveion | 4.882 | 1.51E-18 |
| 14 | 1.14972 | Istamycin C1 | C19 H37 N5 O6 | Positiveion | 4.666 | 2.84E-11 |
| 15 | 1.15362 | Inspra | C24 H30 O6 | Positiveion | 5.612 | 6.75E-10 |
| 16 | 1.13732 | Glipizide | C21 H27 N5 O4 S | Positiveion | 10.291 | 2.12E-09 |
| 17 | 1.00644 | Cyprodenate | C13 H25 N O2 | Positiveion | 7.36 | 6.91E-05 |
| 18 | 1.14519 | Capsidiol | C15 H24 O2 | Positiveion | 6.596 | 6.76E-13 |
| 19 | 1.14786 | 6-[8-Hydroxy-1-(hydroxymethyl)octahydro-2H-quinolizin-3-yl]-2-piperidinone | C15 H26 N2 O3 | Positiveion | 4.729 | 5.83E-11 |
| 20 | 1.15789 | 6,6',7',12'-Tetramethoxy-2,2,2',2'-tetramethyltubocuraran-2,2'-diium | C40 H48 N2 O6 | Positiveion | 8.45 | 9.89E-18 |
| 21 | 1.15884 | 5-Aminopentanamide | C5 H12 N2 O | Positiveion | 4.466 | 1.05E-17 |
| 22 | 1.14784 | 2,3,4,5,6-Pentahydroxy-N-(2-hydroxyethyl)hexanamide | C8 H17 N O7 | Positiveion | 4.978 | 1.12E-11 |
| 23 | 1.03515 | 1-oleoyl-sn-glycero-3-phosphoethanolamine | C23 H46 N O7 P | Positiveion | 9.52 | 5.75E-07 |
| 24 | 1.10359 | 1-Oleoylglycerophosphocholine | C26 H52 N O7 P | Positiveion | 10.106 | 6.66E-07 |
| 25 | 1.11017 | 1-Methylpyrrolinium | C5 H9 N | Positiveion | 0.897 | 4.00E-08 |
| 26 | 1.04802 | 1-Linoleoylglycerophosphocholine | C26 H50 N O7 P | Positiveion | 9.26 | 1.35E-06 |
| 27 | 1.1578 | (4R,5S,6S,7R,9R,10R,11E,13E,16R)-6-{[(2S,3R,4R,5S,6R)-5-{[(2S,4R,5S,6S)-4,5-Dihydroxy-4,6-dimethyltetrahydro-2H-pyran-2-yl]oxy}-4-(dimethylamino)-3-hydroxy-6-methyltetrahydro-2H-pyran-2-yl]oxy}-10-{[( 2R,5S,6R)-5-(dimethylamino)-6-methyltetrahydro-2H-pyran-2-yl]oxy}-5-methoxy-9,16-dimethyl-2-oxo-7-(2-oxoethyl)oxacyclohexadeca-11,13-dien-4-yl acetate | C45 H76 N2 O15 | Positiveion | 5.503 | 2.45E-16 |
| 28 | 1.14652 | (2S,3R)-2-(Dodecanoylamino)-3-hydroxyoctadecyl 5-acetamido-6-[(1S,2R)-2-({5-acetamido-3,5-dideoxy-6-[(1R,2R)-1,2,3-trihydroxypropyl]-beta-L-threo-hex-2-ulopyranonosyl}oxy)-1,3-dihydroxypropyl]-3,5-did eoxy-beta-L-threo-hex-2-ulopyranonosyl-(2->3)-beta-D-galactopyranosyl-(1->4)-beta-D-glucopyranoside | C64 H115 N3 O29 | Positiveion | 8.951 | 5.19E-08 |
| 29 | 1.15939 | (15R,21S)-18,21,24,24-Tetrahydroxy-3-methyl-18,24-dioxido-12-oxo-13,17,19,23-tetraoxa-18lambda~5~,24lambda~5~-diphosphatetracosan-15-yl (9Z,11Z)-9,11-octadecadienoate | C37 H70 O13 P2 | Positiveion | 8.407 | 1.40E-20 |
| 30 | 1.13731 | 4760 | C12 H18 N4 O2 | Positiveion | 5.118 | 1.79E-10 |
| 31 | 1.02602 | 2639 | C14 H24 O2 | Negativeion | 7.38 | 0.027898321 |
| 32 | 1.07614 | 2646 | C15 H26 O2 | Negativeion | 7.772 | 0.027298215 |
| 33 | 1.7043 | 3138020 | C15 H22 O2 | Negativeion | 9.066 | 2.01E-07 |
| 34 | 1.53077 | (+/-)-Camphoric acid | C10 H16 O4 | Negativeion | 5.049 | 5.67E-05 |
| 35 | 1.63671 | (2R)-1-{[(2-Aminoethoxy)(hydroxy)phosphoryl]oxy}-3-hydroxy-2-propanyl (4Z,7Z,10Z,13Z,16Z)-4,7,10,13,16-docosapentaenoate | C27 H46 N O7 P | Negativeion | 8.68 | 0.000116595 |
| 36 | 1.45664 | (3alpha,4beta,5alpha,6alpha,7beta,14beta,22R)-3,4,6,7,29-Pentahydroxy-22,29-epoxystigmastan-15-one | C29 H48 O7 | Negativeion | 10.121 | 0.000342194 |
| 37 | 1.13869 | (5Z_9E_14Z)-(8xi_11R_12S)-11_12-Epoxy-8-hydroxyicosa-5_9_14-trienoicacid | C20 H32 O4 | Negativeion | 8.745 | 0.012229687 |
| 38 | 1.73708 | (6aR_11aR)-3_9-Dihydroxypterocarpan | C15 H12 O4 | Negativeion | 5.985 | 2.54E-06 |
| 39 | 1.05086 | (6aS_11aS)-3_6a_9-Trihydroxypterocarpan | C15 H12 O5 | Negativeion | 6.56 | 0.038872162 |
| 40 | 1.47848 | (6Z)-Octadecenoicacid | C18 H34 O2 | Negativeion | 11.502 | 0.000894314 |
| 41 | 1.53187 | (9Z)-(13S)-12_13-Epoxyoctadeca-9_11-dienoicacid | C18 H30 O3 | Negativeion | 9.492 | 0.000567712 |
| 42 | 1.46422 | (R)-10-Hydroxystearate | C18 H36 O3 | Negativeion | 11.028 | 0.000163642 |
| 43 | 1.49232 | [FA(18:0)]12R_13S-epoxy-9Z-octadecenoicacid | C18 H32 O3 | Negativeion | 9.037 | 0.000215296 |
| 44 | 1.37449 | [FA(18:2)]9Z_11E-octadecadienoicacid | C18 H32 O2 | Negativeion | 9.219 | 0.000829017 |
| 45 | 1.53816 | [FA(20:4)]17R_18S-epoxy-5Z_8Z_11Z_14Z-eicosatetraenoicacid | C20 H30 O3 | Negativeion | 8.897 | 3.67E-05 |
| 46 | 1.6993 | [FA(22:5)]7Z_10Z_13Z_16Z_19Z-docosapentaenoicacid | C22 H34 O2 | Negativeion | 10.971 | 1.01E-06 |
| 47 | 1.49703 | [FAhydroxy(18:0)]12_13-dihydroxy-9Z-octadecenoicacid | C18 H34 O4 | Negativeion | 8.16 | 0.000266315 |
| 48 | 1.81234 | [SThydroxy(3:0)]21-hydroxy-pregn-4-ene-3_11_20-trione | C21 H28 O4 | Negativeion | 8.392 | 5.38E-07 |
| 49 | 1.5109 | [SThydroxy(3:0)]5alpha-pregnan-17alpha_21-dihydroxy-3_11_20-trione | C21 H30 O5 | Negativeion | 9.825 | 0.005170425 |
| 50 | 1.56924 | 1-(4-Methoxyphenyl)-4-methyl-3-oxopentyl hydrogen sulfate | C13 H18 O6 S | Negativeion | 6.407 | 0.000115087 |
| 51 | 1.6861 | 1_7-Dimethylxanthine(paraxanthine) | C7 H8 N4 O2 | Negativeion | 4.149 | 1.69E-07 |
| 52 | 1.42114 | 11beta_17alpha_21-Trihydroxy-5beta-pregnane-3_20-dione | C21 H32 O5 | Negativeion | 9.037 | 0.001158525 |
| 53 | 1.25967 | 12-Hydroxydodecanoicacid | C12 H24 O3 | Negativeion | 8.355 | 0.017583307 |
| 54 | 1.54495 | 13-Hydroxy-9-methoxy-10-oxo-11-octadecenoic acid | C19 H34 O5 | Negativeion | 9.584 | 0.000116905 |
| 55 | 1.0606 | 1D-chiro-inositol | C6 H12 O6 | Negativeion | 0.795 | 0.034561536 |
| 56 | 1.77938 | 2-(3,4-Dihydroxyphenyl)-3,7-dihydroxy-5-chromanesulfinic acid | C15 H14 O7 S | Negativeion | 5.948 | 7.46E-07 |
| 57 | 1.53292 | 2-(5'-methylthio)pentylmalate | C10 H18 O5 S | Negativeion | 5.736 | 0.000184304 |
| 58 | 1.68101 | 2-[(11Z,14Z)-icosadienoyl]-sn-glycero-3-phosphoethanolamine | C25 H48 N O7 P | Negativeion | 9.269 | 5.26E-07 |
| 59 | 1.30804 | 2_4-Dihydroxybenzoicacid | C7 H6 O4 | Negativeion | 5.015 | 0.001365769 |
| 60 | 1.32211 | 2_5-Dihydroxybenzaldehyde | C7 H6 O3 | Negativeion | 5.213 | 0.001615233 |
| 61 | 1.48753 | 2-linoleoyl-sn-glycero-3-phosphoethanolamine | C23 H44 N O7 P | Negativeion | 8.941 | 0.000425451 |
| 62 | 1.30803 | 2-Methylhippuricacid | C10 H11 N O3 | Negativeion | 4.789 | 0.007841874 |
| 63 | 1.61527 | 2-Oxooctadecanoicacid | C18 H34 O3 | Negativeion | 10.368 | 0.000117062 |
| 64 | 1.62365 | 3-(7-Hydroxy-4-oxo-4H-chromen-2-yl)phenyl hydrogen sulfate | C15 H10 O7 S | Negativeion | 5.515 | 0.000226815 |
| 65 | 1.05688 | 3-[2-(3-Hydroxy-5-methoxyphenyl)ethyl]phenyl hydrogen sulfate | C15 H16 O6 S | Negativeion | 5.521 | 0.038358313 |
| 66 | 1.50502 | 3-oxopalmitic acid | C16 H30 O3 | Negativeion | 9.63 | 0.000166597 |
| 67 | 1.58528 | 3-Oxotetradecanoic acid | C14 H26 O3 | Negativeion | 8.897 | 0.00074838 |
| 68 | 1.68059 | 4-ethylphenylsulfonic acid | C8 H10 O4 S | Negativeion | 5.97 | 8.24E-08 |
| 69 | 1.66139 | 4-Sulfobenzoic acid | C7 H6 O5 S | Negativeion | 4.79 | 8.62E-06 |
| 70 | 1.5865 | 4-vinylguaiacol sulfate | C9 H10 O5 S | Negativeion | 5.554 | 1.84E-05 |
| 71 | 1.45107 | 5-[(2E,17E)-19-Hydroxy-2,15,18,24-tetramethyl-25-oxa-7-azatetracyclo[20.2.1.0~6,11~.0~11,16~]pentacosa-2,6,14,17-tetraen-14-yl]-3-methyl-2(5H)-furanone | C32 H45 N O4 | Negativeion | 10.129 | 0.000308276 |
| 72 | 1.24635 | Adenine | C5 H5 N5 | Negativeion | 3.736 | 0.004221525 |
| 73 | 1.54586 | callystatin A | C29 H44 O4 | Negativeion | 9.026 | 0.001520249 |
| 74 | 1.79053 | Carnosol | C20 H26 O4 | Negativeion | 8.072 | 8.61E-09 |
| 75 | 1.45492 | Chaparrin | C20 H28 O7 | Negativeion | 6.497 | 0.008280691 |
| 76 | 1.25673 | Desoxycorticosterone acetate | C23 H32 O4 | Negativeion | 10.684 | 0.022838997 |
| 77 | 1.23403 | DibenzylSuccinate | C18 H18 O4 | Negativeion | 6.675 | 0.001695141 |
| 78 | 1.52833 | GibberellinA14 | C20 H28 O5 | Negativeion | 9.545 | 0.004158832 |
| 79 | 1.02528 | GibberellinA24 | C20 H26 O5 | Negativeion | 6.981 | 0.026295061 |
| 80 | 1.5242 | guaiacol sulfate | C7 H8 O5 S | Negativeion | 4.032 | 0.000248055 |
| 81 | 1.37561 | Hexadecanoicacid | C16 H32 O2 | Negativeion | 11.383 | 0.000276179 |
| 82 | 1.20183 | Indole-3-carboxilic acid-O-sulphate | C9 H7 N O5 S | Negativeion | 4.718 | 0.048519649 |
| 83 | 1.65982 | Lanthionine ketimine | C6 H7 N O4 S | Negativeion | 2.703 | 0.000327727 |
| 84 | 1.52097 | laurilsulfate | C12 H26 O4 S | Negativeion | 9.027 | 4.95E-05 |
| 85 | 1.2238 | Medroxyprogesterone | C22 H32 O3 | Negativeion | 9.217 | 0.011594181 |
| 86 | 1.27347 | paracetamol sulfate | C8 H9 N O5 S | Negativeion | 4.485 | 0.022391558 |
| 87 | 1.42653 | p-cresolsulfatepotassium;p-Cresolsulfate | C7 H8 O4 S | Negativeion | 5.235 | 0.005798193 |
| 88 | 1.69617 | pirsidomine | C17 H22 N4 O3 | Negativeion | 6.218 | 6.62E-06 |
| 89 | 1.26426 | Pseudouridine | C9 H12 N2 O6 | Negativeion | 0.903 | 0.018168044 |
| 90 | 1.15556 | Quillaic Acid | C30 H46 O5 | Negativeion | 9.253 | 0.025505236 |
| 91 | 1.13907 | Thymidine | C10 H14 N2 O5 | Negativeion | 3.817 | 0.037757368 |
| 92 | 1.40435 | Ubiquinone Q4 | C29 H42 O4 | Negativeion | 9.544 | 0.001625092 |
| 93 | 1.18297 | Uricacid | C5 H4 N4 O3 | Negativeion | 0.914 | 0.018860043 |
| 94 | 1.57002 | Xanthoxin;Methoxsalen | C15 H22 O3 | Negativeion | 8.231 | 0.000379183 |
