# Supplementary material for: Exploration of the Characteristics of Intestinal Microbiota and Metabolomics in Different Rat Models of Mongolian Medicine
Source: Evid Based Complement Alternat Med. 2021 Aug 3;2021:5532069. doi: 10.1155/2021/5532069 (PMC8356010; doi:10.1155/2021/5532069)
Supplement: Supplementary Materials — Figure S1: the rarefaction curves of all samples. Table S1: relative abundance of microbial phylum (percentage) in the Heyi rats and control rats. Table S2: relative abundance of microbial phylum (percentage) in the Xila rats and control rats. Table S3: relative abundance of microbial phylum (percentage) in the Badagan rats and control rats. Table S4: differential metabolites of Heyi rat samples compared with control group. Table S5: differential metabolites of Xila rat samples compared with control group. Table S6: differential metabolites of Badagan rat samples compared with control group. Table S7: differential metabolites only present in a group of rats. [file 5532069.f1.zip › 5532069.f1/Table S5-v2.docx]

Table S5 Differential metabolites of Xila rat model samples compared with control group

| **NO** | **VIP** | **Name** | **Formula** | **Mode** | **RT [min]** | **P** |
| --- | --- | --- | --- | --- | --- | --- |
| 1 | 1.10368 | PEG-4 | C8 H18 O5 | positiveion | 3.932 | 8.41E-09 |
| 2 | 1.14884 | PEG n8 | C16 H34 O9 | positiveion | 4.546 | 5.52E-21 |
| 3 | 1.13978 | PEG n7 | C14 H30 O8 | positiveion | 4.132 | 2.01E-18 |
| 4 | 1.13556 | PEG n6 | C12 H26 O7 | positiveion | 4.28 | 3.93E-19 |
| 5 | 1.12463 | PEG n5 | C10 H22 O6 | positiveion | 4.118 | 2.95E-16 |
| 6 | 1.1565 | PEG n16 | C32 H66 O17 | positiveion | 5.233 | 8.56E-23 |
| 7 | 1.15628 | PEG n15 | C30 H62 O16 | positiveion | 5.17 | 3.52E-26 |
| 8 | 1.15786 | PEG n14 | C28 H58 O15 | positiveion | 4.946 | 7.81E-28 |
| 9 | 1.15578 | PEG n13 | C26 H54 O14 | positiveion | 5.015 | 3.97E-25 |
| 10 | 1.15608 | PEG n12 | C24 H50 O13 | positiveion | 4.95 | 3.95E-27 |
| 11 | 1.1551 | PEG n11 | C22 H46 O12 | positiveion | 4.871 | 4.43E-26 |
| 12 | 1.15287 | PEG n10 | C20 H42 O11 | positiveion | 4.558 | 3.82E-24 |
| 13 | 1.13652 | Nootkatone | C15 H22 O | positiveion | 6.604 | 8.22E-18 |
| 14 | 1.15808 | N,N-Diethylthiourea | C5 H12 N2 S | positiveion | 4.882 | 1.47E-18 |
| 15 | 1.03664 | LysoPC(22:5(7Z,10Z,13Z,16Z,19Z)) | C30 H52 N O7 P | positiveion | 9.639 | 0.000340086 |
| 16 | 1.00931 | LysoPC(22:4(7Z,10Z,13Z,16Z)) | C30 H54 N O7 P | positiveion | 10.044 | 3.55E-09 |
| 17 | 1.11206 | LysoPC(20:5(5Z,8Z,11Z,14Z,17Z)) | C28 H48 N O7 P | positiveion | 8.69 | 2.70E-06 |
| 18 | 1.15159 | Istamycin C1 | C19 H37 N5 O6 | positiveion | 4.666 | 5.60E-23 |
| 19 | 1.15448 | Inspra | C24 H30 O6 | positiveion | 5.612 | 3.60E-09 |
| 20 | 1.14419 | Glipizide | C21 H27 N5 O4 S | positiveion | 10.291 | 1.06E-08 |
| 21 | 1.00601 | Diethylene glycol | C4 H10 O3 | positiveion | 0.913 | 3.00E-08 |
| 22 | 1.01326 | Coniferylaldehyde | C10 H10 O3 | positiveion | 5.071 | 9.70E-09 |
| 23 | 1.14843 | Capsidiol | C15 H24 O2 | positiveion | 6.596 | 2.95E-10 |
| 24 | 1.156 | 6-[8-Hydroxy-1-(hydroxymethyl)octahydro-2H-quinolizin-3-yl]-2-piperidinone | C15 H26 N2 O3 | positiveion | 4.505 | 8.71E-26 |
| 25 | 1.14977 | 6,6',7',12'-Tetramethoxy-2,2,2',2'-tetramethyltubocuraran-2,2'-diium | C40 H48 N2 O6 | positiveion | 8.45 | 2.74E-22 |
| 26 | 1.14092 | 5-Aminopentanamide | C5 H12 N2 O | positiveion | 4.466 | 5.42E-21 |
| 27 | 1.11427 | 2-hydroxycaproicacid | C6 H12 O3 | positiveion | 0.917 | 2.38E-13 |
| 28 | 1.15153 | 2,3,4,5,6-Pentahydroxy-N-(2-hydroxyethyl)hexanamide | C8 H17 N O7 | positiveion | 4.978 | 2.37E-09 |
| 29 | 1.12847 | 1-Methylpyrrolinium | C5 H9 N | positiveion | 0.897 | 2.40E-07 |
| 30 | 1.02688 | (R)-3-Amino-2-methylpropanoate | C4 H9 N O2 | positiveion | 0.779 | 2.04E-07 |
| 31 | 1.15195 | (4R,5S,6S,7R,9R,10R,11E,13E,16R)-6-{[(2S,3R,4R,5S,6R)-5-{[(2S,4R,5S,6S)-4,5-Dihydroxy-4,6-dimethyltetrahydro-2H-pyran-2-yl]oxy}-4-(dimethylamino)-3-hydroxy-6-methyltetrahydro-2H-pyran-2-yl]oxy}-10-{[( 2R,5S,6R)-5-(dimethylamino)-6-methyltetrahydro-2H-pyran-2-yl]oxy}-5-methoxy-9,16-dimethyl-2-oxo-7-(2-oxoethyl)oxacyclohexadeca-11,13-dien-4-yl acetate | C45 H76 N2 O15 | positiveion | 5.503 | 9.31E-21 |
| 32 | 1.14554 | (2S,3R)-2-(Dodecanoylamino)-3-hydroxyoctadecyl 5-acetamido-6-[(1S,2R)-2-({5-acetamido-3,5-dideoxy-6-[(1R,2R)-1,2,3-trihydroxypropyl]-beta-L-threo-hex-2-ulopyranonosyl}oxy)-1,3-dihydroxypropyl]-3,5-did eoxy-beta-L-threo-hex-2-ulopyranonosyl-(2->3)-beta-D-galactopyranosyl-(1->4)-beta-D-glucopyranoside | C64 H115 N3 O29 | positiveion | 8.951 | 3.46E-08 |
| 33 | 1.00658 | (2R)-2-Acetoxy-3-[(9Z)-9-octadecen-1-yloxy]propyl 2-(trimethylammonio)ethyl phosphate | C28 H56 N O7 P | positiveion | 10.035 | 4.66E-09 |
| 34 | 1.15434 | (15R,21S)-18,21,24,24-Tetrahydroxy-3-methyl-18,24-dioxido-12-oxo-13,17,19,23-tetraoxa-18lambda~5~,24lambda~5~-diphosphatetracosan-15-yl (9Z,11Z)-9,11-octadecadienoate | C37 H70 O13 P2 | positiveion | 8.407 | 1.68E-25 |
| 35 | 1.1209 | 4760 | C12 H18 N4 O2 | positiveion | 5.118 | 1.76E-10 |
| 36 | 1.03373 | 2639 | C14 H24 O2 | negativeion | 7.38 | 0.030843876 |
| 37 | 1.17645 | 2646 | C15 H26 O2 | negativeion | 7.772 | 0.012421573 |
| 38 | 1.73758 | 3138020 | C15 H22 O2 | negativeion | 9.066 | 5.60E-07 |
| 39 | 1.15291 | (1S,2R,5S)-2-Isopropyl-5-methylcyclohexyl 3-oxobutanoate | C14 H24 O3 | negativeion | 8.389 | 0.002335772 |
| 40 | 1.77871 | (2R)-1-{[(2-Aminoethoxy)(hydroxy)phosphoryl]oxy}-3-hydroxy-2-propanyl (4Z,7Z,10Z,13Z,16Z)-4,7,10,13,16-docosapentaenoate | C27 H46 N O7 P | negativeion | 8.68 | 2.86E-08 |
| 41 | 1.24623 | (5Z_13E)-6_9alpha-Epoxy-11alpha-hydroxy-15-oxoprosta-5_13-dienoate | C20 H30 O5 | negativeion | 8.418 | 0.003921483 |
| 42 | 1.21112 | (5Z_9E_14Z)-(8xi_11R_12S)-11_12-Epoxy-8-hydroxyicosa-5_9_14-trienoicacid | C20 H32 O4 | negativeion | 8.745 | 0.026014053 |
| 43 | 1.74173 | (6aR_11aR)-3_9-Dihydroxypterocarpan | C15 H12 O4 | negativeion | 5.985 | 9.03E-06 |
| 44 | 1.17567 | (9Z)-(13S)-12_13-Epoxyoctadeca-9_11-dienoicacid | C18 H30 O3 | negativeion | 9.492 | 0.000101631 |
| 45 | 1.11963 | (R)(-)-Allantoin | C4 H6 N4 O3 | negativeion | 0.81 | 0.000652284 |
| 46 | 1.33314 | [FA(18:2)]9Z_11E-octadecadienoicacid | C18 H32 O2 | negativeion | 9.219 | 0.000574793 |
| 47 | 1.708 | [FA(20:4)]17R_18S-epoxy-5Z_8Z_11Z_14Z-eicosatetraenoicacid | C20 H30 O3 | negativeion | 8.897 | 4.83E-08 |
| 48 | 1.22594 | [FA(20:4)]5Z_8Z_11Z_14Z-eicosatetraenoicacid | C20 H32 O2 | negativeion | 10.688 | 0.048016796 |
| 49 | 1.75115 | [FA(22:5)]7Z_10Z_13Z_16Z_19Z-docosapentaenoicacid | C22 H34 O2 | negativeion | 10.971 | 8.46E-10 |
| 50 | 1.27167 | [PK]Chrysophanol | C15 H10 O4 | negativeion | 5.909 | 0.039711079 |
| 51 | 1.01823 | [ST(2:0)]22S_25S-furospirost-5-en-3beta_26-diol | C27 H42 O4 | negativeion | 8.912 | 0.002868527 |
| 52 | 1.80506 | [SThydroxy(3:0)]21-hydroxy-pregn-4-ene-3_11_20-trione | C21 H28 O4 | negativeion | 8.392 | 6.38E-07 |
| 53 | 1.56613 | [SThydroxy(3:0)]5alpha-pregnan-17alpha_21-dihydroxy-3_11_20-trione | C21 H30 O5 | negativeion | 9.825 | 0.005185751 |
| 54 | 1.25013 | 1(2H)-Isoquinolinone | C9 H7 N O | negativeion | 5.619 | 0.004082227 |
| 55 | 1.59385 | 13-Hydroxy-9-methoxy-10-oxo-11-octadecenoic acid | C19 H34 O5 | negativeion | 9.584 | 9.53E-10 |
| 56 | 1.16925 | 1D-chiro-inositol | C6 H12 O6 | negativeion | 0.795 | 0.006379218 |
| 57 | 1.367 | 1-stearoyl-sn-glycero-3-phosphoethanolamine | C23 H48 N O7 P | negativeion | 10.332 | 3.83E-06 |
| 58 | 1.78621 | 2-(3,4-Dihydroxyphenyl)-3,7-dihydroxy-5-chromanesulfinic acid | C15 H14 O7 S | negativeion | 5.948 | 1.58E-06 |
| 59 | 1.67926 | 3-(7-Hydroxy-4-oxo-4H-chromen-2-yl)phenyl hydrogen sulfate | C15 H10 O7 S | negativeion | 5.515 | 0.000162835 |
| 60 | 1.41835 | 3-[2-(3-Hydroxy-5-methoxyphenyl)ethyl]phenyl hydrogen sulfate | C15 H16 O6 S | negativeion | 5.521 | 0.000375903 |
| 61 | 1.01623 | 3-Ureidoisobutyrate | C5 H10 N2 O3 | negativeion | 0.781 | 0.00256467 |
| 62 | 1.43467 | 4-(2-Hydroxyethyl)phenyl hydrogen sulfate | C8 H10 O5 S | negativeion | 5.838 | 0.000900972 |
| 63 | 1.00508 | 4-Ethyl-2-methoxyphenyl hydrogen sulfate | C9 H12 O5 S | negativeion | 6.488 | 0.017678414 |
| 64 | 1.7651 | 4-ethylphenylsulfonic acid | C8 H10 O4 S | negativeion | 5.97 | 2.80E-06 |
| 65 | 1.12789 | 4-vinylphenol sulfate | C8 H8 O4 S | negativeion | 5.756 | 0.002582955 |
| 66 | 1.0348 | alpha-L-Arabinose | C5 H10 O5 | negativeion | 0.806 | 0.00227828 |
| 67 | 1.62983 | callystatin A | C29 H44 O4 | negativeion | 9.026 | 0.001536545 |
| 68 | 1.51125 | Carnosol | C20 H26 O4 | negativeion | 8.072 | 9.76E-09 |
| 69 | 1.5119 | Chaparrin | C20 H28 O7 | negativeion | 6.497 | 0.008798028 |
| 70 | 1.26363 | Desoxycorticosterone acetate | C23 H32 O4 | negativeion | 10.684 | 0.020533415 |
| 71 | 1.41779 | DibenzylSuccinate | C18 H18 O4 | negativeion | 6.675 | 2.44E-05 |
| 72 | 1.10184 | Ethyl (2E,4E,6E,8E,10E,12E,14Z,16E)-2,6,11,15-tetramethyl-17-(2,6,6-trimethyl-1-cyclohexen-1-yl)-2,4,6,8,10,12,14,16-heptadecaoctaenoate | C32 H44 O2 | negativeion | 11.948 | 0.00183307 |
| 73 | 1.21172 | GibberellinA12 | C20 H28 O4 | negativeion | 8.417 | 0.00348229 |
| 74 | 1.56939 | GibberellinA14 | C20 H28 O5 | negativeion | 9.545 | 0.005214591 |
| 75 | 1.67771 | guaiacol sulfate | C7 H8 O5 S | negativeion | 4.032 | 0.000129502 |
| 76 | 1.49276 | Indole-3-carboxilic acid-O-sulphate | C9 H7 N O5 S | negativeion | 4.718 | 0.000298375 |
| 77 | 1.53508 | Indoleacrylicacid | C11 H9 N O2 | negativeion | 6.079 | 0.000874567 |
| 78 | 1.0413 | Indoxylsulfate | C8 H7 N O4 S | negativeion | 5.609 | 0.030640525 |
| 79 | 1.6822 | Lanthionine ketimine | C6 H7 N O4 S | negativeion | 2.703 | 0.000391141 |
| 80 | 1.1767 | lysophosphatidylethanolamine (22:6(4Z,7Z,10Z,13Z,16Z,19Z)/0:0) | C27 H44 N O7 P | negativeion | 8.863 | 0.002731567 |
| 81 | 1.44316 | Medroxyprogesterone | C22 H32 O3 | negativeion | 9.217 | 0.00036189 |
| 82 | 1.52323 | paracetamol sulfate | C8 H9 N O5 S | negativeion | 4.485 | 0.007317899 |
| 83 | 1.38245 | p-cresolsulfatepotassium;p-Cresolsulfate | C7 H8 O4 S | negativeion | 5.235 | 0.016207325 |
| 84 | 1.30165 | Taxa-4(20)_11(12)-dien-5alpha-acetoxy-10beta-ol | C22 H34 O3 | negativeion | 9.606 | 0.002392514 |
| 85 | 1.65003 | Ubiquinone Q4 | C29 H42 O4 | negativeion | 9.544 | 0.000619542 |
| 86 | 1.63546 | Xanthoxin;Methoxsalen | C15 H22 O3 | negativeion | 8.231 | 7.64E-05 |
