# Supplementary material for: Exploration of the Characteristics of Intestinal Microbiota and Metabolomics in Different Rat Models of Mongolian Medicine
Source: Evid Based Complement Alternat Med. 2021 Aug 3;2021:5532069. doi: 10.1155/2021/5532069 (PMC8356010; doi:10.1155/2021/5532069)
Supplement: Supplementary Materials — Figure S1: the rarefaction curves of all samples. Table S1: relative abundance of microbial phylum (percentage) in the Heyi rats and control rats. Table S2: relative abundance of microbial phylum (percentage) in the Xila rats and control rats. Table S3: relative abundance of microbial phylum (percentage) in the Badagan rats and control rats. Table S4: differential metabolites of Heyi rat samples compared with control group. Table S5: differential metabolites of Xila rat samples compared with control group. Table S6: differential metabolites of Badagan rat samples compared with control group. Table S7: differential metabolites only present in a group of rats. [file 5532069.f1.zip › 5532069.f1/Table S6-v2.docx]

Table S6 Differential metabolites of Badagan rat model samples compared with control group

| **NO** | **VIP** | **Name** | **Formula** | **Model** | **RT [min]** | **P** |
| --- | --- | --- | --- | --- | --- | --- |
| 1 | 1.03836 | stearoylcarnitine | C25 H49 N O4 | positiveion | 10.843 | 6.87E-05 |
| 2 | 1.09677 | PEG-4 | C8 H18 O5 | positiveion | 0.919 | 4.39E-08 |
| 3 | 1.14283 | PEG n8 | C16 H34 O9 | positiveion | 4.546 | 1.88E-13 |
| 4 | 1.14351 | PEG n7 | C14 H30 O8 | positiveion | 4.42 | 3.06E-14 |
| 5 | 1.13279 | PEG n6 | C12 H26 O7 | positiveion | 4.28 | 2.31E-09 |
| 6 | 1.12416 | PEG n5 | C10 H22 O6 | positiveion | 4.118 | 3.59E-09 |
| 7 | 1.14653 | PEG n16 | C32 H66 O17 | positiveion | 5.233 | 1.51E-17 |
| 8 | 1.14234 | PEG n15 | C30 H62 O16 | positiveion | 5.023 | 1.70E-10 |
| 9 | 1.14569 | PEG n14 | C28 H58 O15 | positiveion | 5.102 | 5.00E-15 |
| 10 | 1.14623 | PEG n13 | C26 H54 O14 | positiveion | 5.027 | 7.17E-17 |
| 11 | 1.1455 | PEG n12 | C24 H50 O13 | positiveion | 4.95 | 4.21E-14 |
| 12 | 1.14531 | PEG n11 | C22 H46 O12 | positiveion | 4.871 | 5.70E-13 |
| 13 | 1.14561 | PEG n10 | C20 H42 O11 | positiveion | 4.558 | 7.06E-16 |
| 14 | 1.12298 | Nootkatone | C15 H22 O | positiveion | 6.604 | 7.59E-09 |
| 15 | 1.14656 | N,N-Diethylthiourea | C5 H12 N2 S | positiveion | 4.882 | 2.25E-17 |
| 16 | 1.14453 | Istamycin C1 | C19 H37 N5 O6 | positiveion | 4.435 | 7.38E-16 |
| 17 | 1.14003 | Inspra | C24 H30 O6 | positiveion | 5.612 | 1.01E-09 |
| 18 | 1.12397 | Glipizide | C21 H27 N5 O4 S | positiveion | 10.291 | 5.81E-10 |
| 19 | 1.07245 | Diethylene glycol | C4 H10 O3 | positiveion | 0.913 | 1.47E-07 |
| 20 | 1.03751 | Creatine | C4 H9 N3 O2 | positiveion | 0.809 | 2.98E-06 |
| 21 | 1.133 | Capsidiol | C15 H24 O2 | positiveion | 6.596 | 2.15E-12 |
| 22 | 1.14132 | 6-[8-Hydroxy-1-(hydroxymethyl)octahydro-2H-quinolizin-3-yl]-2-piperidinone | C15 H26 N2 O3 | positiveion | 4.729 | 1.54E-11 |
| 23 | 1.14273 | 6,6',7',12'-Tetramethoxy-2,2,2',2'-tetramethyltubocuraran-2,2'-diium | C40 H48 N2 O6 | positiveion | 8.45 | 2.43E-14 |
| 24 | 1.14127 | 5-Aminopentanamide | C5 H12 N2 O | positiveion | 4.466 | 1.26E-11 |
| 25 | 1.03793 | 3-hydroxyhexadecanoylcarnitine | C23 H45 N O5 | positiveion | 8.667 | 0.000152784 |
| 26 | 1.10213 | 3,5a,9-Trimethyl-2,3,3a,4,5,5a,8,9b-octahydronaphtho[1,2-b]furan-2,8-dione | C15 H18 O3 | positiveion | 6.691 | 1.20E-07 |
| 27 | 1.08279 | 2-hydroxycaproicacid | C6 H12 O3 | positiveion | 0.917 | 2.70E-07 |
| 28 | 1.13696 | 2,3,4,5,6-Pentahydroxy-N-(2-hydroxyethyl)hexanamide | C8 H17 N O7 | positiveion | 4.978 | 7.57E-11 |
| 29 | 1.00271 | 1-Oleoylglycerophosphocholine | C26 H52 N O7 P | positiveion | 10.106 | 6.41E-05 |
| 30 | 1.09387 | 1-Methylpyrrolinium | C5 H9 N | positiveion | 0.897 | 4.83E-08 |
| 31 | 1.0257 | Valerolactam | C5 H9 N O | positiveion | 0.917 | 7.55E-06 |
| 32 | 1.07999 | (R)-3-Amino-2-methylpropanoate | C4 H9 N O2 | positiveion | 0.779 | 4.28E-07 |
| 33 | 1.02546 | (4S)-4-{[(9Z)-3-Hydroxy-9-hexadecenoyl]oxy}-4-(trimethylammonio)butanoate | C23 H43 N O5 | positiveion | 8.154 | 9.10E-05 |
| 34 | 1.14512 | (4R,5S,6S,7R,9R,10R,11E,13E,16R)-6-{[(2S,3R,4R,5S,6R)-5-{[(2S,4R,5S,6S)-4,5-Dihydroxy-4,6-dimethyltetrahydro-2H-pyran-2-yl]oxy}-4-(dimethylamino)-3-hydroxy-6-methyltetrahydro-2H-pyran-2-yl]oxy}-10-{[( 2R,5S,6R)-5-(dimethylamino)-6-methyltetrahydro-2H-pyran-2-yl]oxy}-5-methoxy-9,16-dimethyl-2-oxo-7-(2-oxoethyl)oxacyclohexadeca-11,13-dien-4-yl acetate | C45 H76 N2 O15 | positiveion | 5.503 | 2.39E-15 |
| 35 | 1.12906 | (2S,3R)-2-(Dodecanoylamino)-3-hydroxyoctadecyl 5-acetamido-6-[(1S,2R)-2-({5-acetamido-3,5-dideoxy-6-[(1R,2R)-1,2,3-trihydroxypropyl]-beta-L-threo-hex-2-ulopyranonosyl}oxy)-1,3-dihydroxypropyl]-3,5-did eoxy-beta-L-threo-hex-2-ulopyranonosyl-(2->3)-beta-D-galactopyranosyl-(1->4)-beta-D-glucopyranoside | C64 H115 N3 O29 | positiveion | 8.951 | 6.07E-08 |
| 36 | 1.14476 | (15R,21S)-18,21,24,24-Tetrahydroxy-3-methyl-18,24-dioxido-12-oxo-13,17,19,23-tetraoxa-18lambda~5~,24lambda~5~-diphosphatetracosan-15-yl (9Z,11Z)-9,11-octadecadienoate | C37 H70 O13 P2 | positiveion | 8.407 | 9.49E-15 |
| 37 | 1.12724 | 4760 | C12 H18 N4 O2 | positiveion | 5.118 | 2.88E-10 |
| 38 | 1.04461 | (5Z_13E)-6_9alpha-Epoxy-11alpha-hydroxy-15-oxoprosta-5_13-dienoate | C20 H30 O5 | negativeion | 8.418 | 0.023571868 |
| 39 | 1.19502 | paracetamol sulfate | C8 H9 N O5 S | negativeion | 4.485 | 0.028995087 |
| 40 | 1.27657 | Chaparrin | C20 H28 O7 | negativeion | 6.497 | 0.009465373 |
| 41 | 1.55336 | (6aR_11aR)-3_9-Dihydroxypterocarpan | C15 H12 O4 | negativeion | 5.985 | 1.29E-05 |
| 42 | 1.15101 | 4-vinylguaiacol sulfate | C9 H10 O5 S | negativeion | 6.154 | 0.010701615 |
| 43 | 1.41682 | 3-oxopalmitic acid | C16 H30 O3 | negativeion | 9.63 | 0.000112904 |
| 44 | 1.15632 | Cucurbitacin S | C30 H42 O6 | negativeion | 8.912 | 0.003382063 |
| 45 | 1.10676 | Palmitoleicacid | C16 H30 O2 | negativeion | 10.675 | 0.015444306 |
| 46 | 1.02839 | 3-[2-(3-Hydroxy-5-methoxyphenyl)ethyl]phenyl hydrogen sulfate | C15 H16 O6 S | negativeion | 5.521 | 0.014185705 |
| 47 | 1.59156 | [SThydroxy(3:0)]21-hydroxy-pregn-4-ene-3_11_20-trione | C21 H28 O4 | negativeion | 8.392 | 5.74E-07 |
| 48 | 1.28226 | 12-deoxyphorbol 20-acetate 13-(2-methylbutanoate) | C27 H38 O7 | negativeion | 7.329 | 0.0012111 |
| 49 | 1.097 | Pseudouridine | C9 H12 N2 O6 | negativeion | 0.903 | 0.043561495 |
| 50 | 1.31387 | 3-Oxododecanoicacid | C12 H22 O3 | negativeion | 7.87 | 0.004109914 |
| 51 | 1.08438 | Desoxycorticosterone acetate | C23 H32 O4 | negativeion | 10.684 | 0.0268344 |
| 52 | 1.30141 | Ethyl (2E,4E,6E,8E,10E,12E,14Z,16E)-2,6,11,15-tetramethyl-17-(2,6,6-trimethyl-1-cyclohexen-1-yl)-2,4,6,8,10,12,14,16-heptadecaoctaenoate | C32 H44 O2 | negativeion | 11.948 | 0.000876341 |
| 53 | 1.01953 | Hexadecanoicacid | C16 H32 O2 | negativeion | 11.383 | 0.014244336 |
| 54 | 1.27285 | Indoleacrylicacid | C11 H9 N O2 | negativeion | 6.079 | 0.002546107 |
| 55 | 1.0808 | 3-Methylindole | C9 H9 N | negativeion | 6.242 | 0.007063072 |
| 56 | 1.05512 | GibberellinA12 | C20 H28 O4 | negativeion | 8.417 | 0.022113551 |
| 57 | 1.44043 | 3-(7-Hydroxy-4-oxo-4H-chromen-2-yl)phenyl hydrogen sulfate | C15 H10 O7 S | negativeion | 5.515 | 0.000335508 |
| 58 | 1.37015 | Lanthionine ketimine | C6 H7 N O4 S | negativeion | 2.703 | 0.001430886 |
| 59 | 1.25706 | 12-Hydroxydodecanoicacid | C12 H24 O3 | negativeion | 8.355 | 0.003857477 |
| 60 | 1.01185 | (6aS_11aS)-3_6a_9-Trihydroxypterocarpan | C15 H12 O5 | negativeion | 6.56 | 0.029112854 |
| 61 | 1.07112 | 2646 | C15 H26 O2 | negativeion | 7.772 | 0.020544779 |
| 62 | 1.07823 | 2639 | C14 H24 O2 | negativeion | 7.38 | 0.009050273 |
| 63 | 1.52352 | 13-Hydroxy-9-methoxy-10-oxo-11-octadecenoic acid | C19 H34 O5 | negativeion | 9.584 | 2.66E-05 |
| 64 | 1.58883 | 2-(3,4-Dihydroxyphenyl)-3,7-dihydroxy-5-chromanesulfinic acid | C15 H14 O7 S | negativeion | 5.948 | 1.62E-06 |
| 65 | 1.32001 | [SThydroxy(3:0)]5alpha-pregnan-17alpha_21-dihydroxy-3_11_20-trione | C21 H30 O5 | negativeion | 9.825 | 0.005200681 |
| 66 | 1.08694 | Bis(2-ethylhexyl)phthalate | C24 H38 O4 | negativeion | 7.725 | 0.026888286 |
| 67 | 1.22288 | [PK]Chrysophanol | C15 H10 O4 | negativeion | 5.909 | 0.018729484 |
| 68 | 1.40212 | 2_4-Dihydroxybenzoicacid | C7 H6 O4 | negativeion | 5.015 | 0.00034216 |
| 69 | 1.192 | 2'-Hydroxydaidzein | C15 H10 O5 | negativeion | 6.489 | 0.028423795 |
| 70 | 1.1202 | Medroxyprogesterone | C22 H32 O3 | negativeion | 9.217 | 0.017533491 |
| 71 | 1.18502 | [ST(2:0)]22S_25S-furospirost-5-en-3beta_26-diol | C27 H42 O4 | negativeion | 8.912 | 0.002497563 |
| 72 | 1.48368 | (2R)-1-{[(2-Aminoethoxy)(hydroxy)phosphoryl]oxy}-3-hydroxy-2-propanyl (4Z,7Z,10Z,13Z,16Z)-4,7,10,13,16-docosapentaenoate | C27 H46 N O7 P | negativeion | 8.68 | 0.0001946 |
| 73 | 1.16159 | Thymidine | C10 H14 N2 O5 | negativeion | 3.817 | 0.010563996 |
| 74 | 1.13618 | 1-stearoyl-sn-glycero-3-phosphoethanolamine | C23 H48 N O7 P | negativeion | 10.332 | 0.005272864 |
| 75 | 1.15798 | 1-arachidonoyl-sn-glycero-3-phosphoethanolamine | C25 H44 N O7 P | negativeion | 8.918 | 0.002687561 |
| 76 | 1.30556 | p-cresolsulfatepotassium;p-Cresolsulfate | C7 H8 O4 S | negativeion | 5.235 | 0.003759795 |
| 77 | 1.56521 | 4-ethylphenylsulfonic acid | C8 H10 O4 S | negativeion | 5.97 | 4.25E-07 |
| 78 | 1.20769 | 1D-chiro-inositol | C6 H12 O6 | negativeion | 0.795 | 0.003306797 |
| 79 | 1.53157 | 2-[(11Z,14Z)-icosadienoyl]-sn-glycero-3-phosphoethanolamine | C25 H48 N O7 P | negativeion | 9.269 | 7.00E-07 |
| 80 | 1.1265 | (2R)-1-{[(2-Aminoethoxy)(hydroxy)phosphoryl]oxy}-3-hydroxy-2-propanyl (7Z,10Z,13Z,16Z)-7,10,13,16-docosatetraenoate | C27 H48 N O7 P | negativeion | 9.171 | 0.020092573 |
| 81 | 1.24853 | Indoxylsulfate | C8 H7 N O4 S | negativeion | 4.731 | 0.002492622 |
| 82 | 1.53249 | Carnosol | C20 H26 O4 | negativeion | 8.072 | 2.64E-06 |
| 83 | 1.33443 | 2-linoleoyl-sn-glycero-3-phosphoethanolamine | C23 H44 N O7 P | negativeion | 8.941 | 0.000852678 |
| 84 | 1.3965 | 4-vinylphenol sulfate | C8 H8 O4 S | negativeion | 5.756 | 5.36E-05 |
| 85 | 1.48781 | (9Z)-(13S)-12_13-Epoxyoctadeca-9_11-dienoicacid | C18 H30 O3 | negativeion | 9.492 | 1.05E-05 |
| 86 | 1.41139 | 3-Oxotetradecanoic acid | C14 H26 O3 | negativeion | 8.897 | 0.000501087 |
| 87 | 1.06514 | 3-Ureidoisobutyrate | C5 H10 N2 O3 | negativeion | 0.781 | 0.009741424 |
| 88 | 1.54925 | 3138020 | C15 H22 O2 | negativeion | 9.066 | 2.04E-07 |
| 89 | 1.50857 | Indole-3-carboxilic acid-O-sulphate | C9 H7 N O5 S | negativeion | 4.718 | 1.34E-06 |
| 90 | 1.05329 | alpha-L-Arabinose | C5 H10 O5 | negativeion | 0.806 | 0.014495744 |
| 91 | 1.4898 | 4-(2-Hydroxyethyl)phenyl hydrogen sulfate | C8 H10 O5 S | negativeion | 5.838 | 8.06E-06 |
| 92 | 1.36838 | callystatin A | C29 H44 O4 | negativeion | 9.026 | 0.00198473 |
| 93 | 1.23034 | (6Z)-Octadecenoicacid | C18 H34 O2 | negativeion | 11.502 | 0.006177204 |
| 94 | 1.32654 | GibberellinA14 | C20 H28 O5 | negativeion | 9.545 | 0.004770436 |
| 95 | 1.35497 | [FA(20:4)]17R_18S-epoxy-5Z_8Z_11Z_14Z-eicosatetraenoicacid | C20 H30 O3 | negativeion | 8.897 | 0.000269724 |
| 96 | 1.22652 | (1S,2R,5S)-2-Isopropyl-5-methylcyclohexyl 3-oxobutanoate | C14 H24 O3 | negativeion | 8.389 | 0.012868882 |
| 97 | 1.40974 | 2-Oxooctadecanoicacid | C18 H34 O3 | negativeion | 10.368 | 0.000298706 |
| 98 | 1.57021 | DibenzylSuccinate | C18 H18 O4 | negativeion | 6.675 | 5.06E-08 |
| 99 | 1.33036 | Ubiquinone Q4 | C29 H42 O4 | negativeion | 9.544 | 0.001014738 |
| 100 | 1.51956 | [FA(22:5)]7Z_10Z_13Z_16Z_19Z-docosapentaenoicacid | C22 H34 O2 | negativeion | 10.971 | 1.45E-06 |
