# Supplementary material for: Exploration of the Characteristics of Intestinal Microbiota and Metabolomics in Different Rat Models of Mongolian Medicine
Source: Evid Based Complement Alternat Med. 2021 Aug 3;2021:5532069. doi: 10.1155/2021/5532069 (PMC8356010; doi:10.1155/2021/5532069)
Supplement: Supplementary Materials — Figure S1: the rarefaction curves of all samples. Table S1: relative abundance of microbial phylum (percentage) in the Heyi rats and control rats. Table S2: relative abundance of microbial phylum (percentage) in the Xila rats and control rats. Table S3: relative abundance of microbial phylum (percentage) in the Badagan rats and control rats. Table S4: differential metabolites of Heyi rat samples compared with control group. Table S5: differential metabolites of Xila rat samples compared with control group. Table S6: differential metabolites of Badagan rat samples compared with control group. Table S7: differential metabolites only present in a group of rats. [file 5532069.f1.zip › 5532069.f1/Table S7-v2.docx]

Table 1 Differential metabolites only present in a group of rats

| **Name** | **Formula** | **RT [min]** | **Group** |
| --- | --- | --- | --- |
| Cyprodenate | C13 H25 N O2 | 7.36 | Heyi rat model |
| 1-oleoyl-sn-glycero-3-phosphoethanolamine | C23 H46 N O7 P | 9.52 | Heyi rat model |
| 1-Linoleoylglycerophosphocholine | C26 H50 N O7 P | 9.26 | Heyi rat model |
| (+/-)-Camphoric acid | C10 H16 O4 | 5.049 | Heyi rat model |
| (3alpha,4beta,5alpha,6alpha,7beta,14beta,22R)-3,4,6,7,29-Pentahydroxy-22,29-epoxystigmastan-15-one | C29 H48 O7 | 10.121 | Heyi rat model |
| (R)-10-Hydroxystearate | C18 H36 O3 | 11.028 | Heyi rat model |
| [FA(18:0)]12R_13S-epoxy-9Z-octadecenoicacid | C18 H32 O3 | 9.037 | Heyi rat model |
| [FAhydroxy(18:0)]12_13-dihydroxy-9Z-octadecenoicacid | C18 H34 O4 | 8.16 | Heyi rat model |
| 1-(4-Methoxyphenyl)-4-methyl-3-oxopentyl hydrogen sulfate | C13 H18 O6 S | 6.407 | Heyi rat model |
| 1_7-Dimethylxanthine(paraxanthine) | C7 H8 N4 O2 | 4.149 | Heyi rat model |
| 11beta_17alpha_21-Trihydroxy-5beta-pregnane-3_20-dione | C21 H32 O5 | 9.037 | Heyi rat model |
| 2-(5'-methylthio)pentylmalate | C10 H18 O5 S | 5.736 | Heyi rat model |
| 2_5-Dihydroxybenzaldehyde | C7 H6 O3 | 5.213 | Heyi rat model |
| 2-Methylhippuricacid | C10 H11 N O3 | 4.789 | Heyi rat model |
| 4-Sulfobenzoic acid | C7 H6 O5 S | 4.79 | Heyi rat model |
| 5-[(2E,17E)-19-Hydroxy-2,15,18,24-tetramethyl-25-oxa-7-azatetracyclo[20.2.1.0~6,11~.0~11,16~]pentacosa-2,6,14,17-tetraen-14-yl]-3-methyl-2(5H)-furanone | C32 H45 N O4 | 10.129 | Heyi rat model |
| Adenine | C5 H5 N5 | 3.736 | Heyi rat model |
| GibberellinA24 | C20 H26 O5 | 6.981 | Heyi rat model |
| laurilsulfate | C12 H26 O4 S | 9.027 | Heyi rat model |
| pirsidomine | C17 H22 N4 O3 | 6.218 | Heyi rat model |
| Quillaic Acid | C30 H46 O5 | 9.253 | Heyi rat model |
| Uricacid | C5 H4 N4 O3 | 0.914 | Heyi rat model |
| LysoPC(22:5(7Z,10Z,13Z,16Z,19Z)) | C30 H52 N O7 P | 9.639 | Xila rat model |
| LysoPC(22:4(7Z,10Z,13Z,16Z)) | C30 H54 N O7 P | 10.044 | Xila rat model |
| LysoPC(20:5(5Z,8Z,11Z,14Z,17Z)) | C28 H48 N O7 P | 8.69 | Xila rat model |
| Coniferylaldehyde | C10 H10 O3 | 5.071 | Xila rat model |
| (2R)-2-Acetoxy-3-[(9Z)-9-octadecen-1-yloxy]propyl 2-(trimethylammonio)ethyl phosphate | C28 H56 N O7 P | 10.035 | Xila rat model |
| (R)(-)-Allantoin | C4 H6 N4 O3 | 0.81 | Xila rat model |
| [FA(20:4)]5Z_8Z_11Z_14Z-eicosatetraenoicacid | C20 H32 O2 | 10.688 | Xila rat model |
| 1(2H)-Isoquinolinone | C9 H7 N O | 5.619 | Xila rat model |
| 4-Ethyl-2-methoxyphenyl hydrogen sulfate | C9 H12 O5 S | 6.488 | Xila rat model |
| lysophosphatidylethanolamine (22:6(4Z,7Z,10Z,13Z,16Z,19Z)/0:0) | C27 H44 N O7 P | 8.863 | Xila rat model |
| Taxa-4(20)_11(12)-dien-5alpha-acetoxy-10beta-ol | C22 H34 O3 | 9.606 | Xila rat model |
| stearoylcarnitine | C25 H49 N O4 | 10.843 | Badagan rat model |
| Creatine | C4 H9 N3 O2 | 0.809 | Badagan rat model |
| 3-hydroxyhexadecanoylcarnitine | C23 H45 N O5 | 8.667 | Badagan rat model |
| 3,5a,9-Trimethyl-2,3,3a,4,5,5a,8,9b-octahydronaphtho[1,2-b]furan-2,8-dione | C15 H18 O3 | 6.691 | Badagan rat model |
| Valerolactam | C5 H9 N O | 0.917 | Badagan rat model |
| (4S)-4-{[(9Z)-3-Hydroxy-9-hexadecenoyl]oxy}-4-(trimethylammonio)butanoate | C23 H43 N O5 | 8.154 | Badagan rat model |
| Cucurbitacins | C30 H42 O6 | 8.912 | Badagan rat model |
| Palmitoleicacid | C16 H30 O2 | 10.675 | Badagan rat model |
| 12-deoxyphorbol 20-acetate 13-(2-methylbutanoate) | C27 H38 O7 | 7.329 | Badagan rat model |
| 3-Oxododecanoicacid | C12 H22 O3 | 7.87 | Badagan rat model |
| 3-Methylindole | C9 H9 N | 6.242 | Badagan rat model |
| Bis(2-ethylhexyl)phthalate | C24 H38 O4 | 7.725 | Badagan rat model |
| 2'-Hydroxydaidzein | C15 H10 O5 | 6.489 | Badagan rat model |
| 1-arachidonoyl-sn-glycero-3-phosphoethanolamine | C25 H44 N O7 P | 8.918 | Badagan rat model |
| (2R)-1-{[(2-Aminoethoxy)(hydroxy)phosphoryl]oxy}-3-hydroxy-2-propanyl (7Z,10Z,13Z,16Z)-7,10,13,16-docosatetraenoate | C27 H48 N O7 P | 9.171 | Badagan rat model |
